# Taxonomic revision of the African genus *Greenwayodendron* (Annonaceae)

**DOI:** 10.3897/phytokeys.114.27395

**Published:** 2018-12-31

**Authors:** Brandet-Junior Lissambou, Olivier J. Hardy, Christiane Atteke, Tariq Stevart, Gilles Dauby, Bertrand Mbatchi, Bonaventure Sonke, Thomas L.P. Couvreur

**Affiliations:** 1 Département de Biologie, Faculté des Sciences, Université des Sciences et Techniques de Masuku (USTM). BP 943, Franceville, Gabon; 2 Evolutionary Biology and Ecology Unit, Faculté des Sciences, Université Libre de Bruxelles, 50 Av. F. Roosevelt, CP 160/12, B-1050 Brussels, Belgium; 3 Herbarium and Library of African Botany, Faculté des Sciences, Université Libre de Bruxelles, Boulevard du Triomphe, CP265, B-1050 Brussels, Belgium; 4 Plant Systematics and Ecology Laboratory, Higher Teachers Training College, University of Yaoundé I, BP 047, Yaoundé, Cameroon; 5 Institut de Recherche pour le Développement (IRD), Université Montpellier, UMR DIADE, BP 64501, F-34394 Montpellier cedex 5, France; 6 Naturalis Biodiversity Centre, Botany Section, Darwinweg 2, 2333 CR Leiden, The Netherlands

**Keywords:** Annonaceae, *
Greenwayodendron
*, tree, rainforest, new species

## Abstract

*Greenwayodendron* (Annonaceae) is a tropical African genus of trees occurring mainly in rain forests. Until recently, *Greenwayodendron* contained only two species: *Greenwayodendronoliveri* from West Africa and *Greenwayodendronsuaveolens* from Central and East Africa.

Genetic data, using chloroplast haplotypes and nuclear microsatellites as well as morphometric analyses, provided important information on the delineation of species. *Greenwayodendron* now contains six species, including two new species (*Greenwayodendronglabrum* Lissambou, Hardy & Couvreur, **sp. nov.** and *Greenwayodendronlittorale* Lissambou, Dauby & Couvreur, **sp. nov.**). Greenwayodendronsuaveolensvar.gabonica and Greenwayodendronsuaveolenssubsp.usambaricum are recognised as distinct species: *Greenwayodendrongabonicum***comb. nov.** and *Greenwayodendronusambaricum***comb. nov.**, respectively. A key, detailed descriptions of morphology and geographic distributions, as well as notes on their ecology and uses are presented for all species. Preliminary conservation assessments following IUCN criteria are also provided. Two species are preliminarily identified as threatened, one as Endangered and one as Vulnerable.

## Introduction

The family Annonaceae (Magnoliidae) comprises trees, shrubs and lianas (APG 2003; Chatrou et al. 2012; Couvreur et al. 2015) that play a key ecological role in tropical forests (Gentry 1993). In continental Africa (excluding Madagascar), 42 genera and around 400 species have been recorded to date (Couvreur et al. 2011; Couvreur et al. 2012; Ghogue et al. 2017). During the last decade, several revisions and monographs have significantly improved our understanding of African Annonaceae such as *Hexalobus* (Botermans et al. 2011), *Monodora* and *Isolona* (Couvreur 2009), *Uvariastrum* (Couvreur 2014), *Neostenanthera* (Fero et al. 2014), *Brieya* and *Piptostigma* (Ghogue et al. 2017), *Annickia* (Versteegh and Sosef 2007) and *Duguetia* (Maas et al. 2003).

The tribe Piptostigmateae (Malmeoideae) contains seven African genera: *Annickia* Setten & Maas, *Brieya* De Wild., *Greenwayodendron* Verdc., *Mwasumbia* Couvreur & Johnson, *Piptostigma* Oliv., *Polyceratocarpus* Engl. & Diels and *Sirdavidia* Couvreur & Sauquet (Couvreur et al. 2009; Chatrou et al. 2012; Couvreur et al. 2015; Ghogue et al. 2017). *Greenwayodendron* is inferred with strong support as sister to a clade containing the genera *Mwasumbia*, *Sirdavidia*, *Brieya*, *Polyceratocarpus* and *Piptostigma* (Couvreur et al. 2015; Ghogue et al. 2017).

*Greenwayodendron* contains two species (Table 1), namely *G.oliveri* (Engl) Verdc. in West Africa and *G.suaveolens* (Engl. & Diels) Verdc. in Central Africa (Le Thomas 1969; Verdcourt 1969). *Greenwayodendronsuaveolens* was further divided into two subspecies (subsp. usambaricum Verdc. and subsp. suaveolens Verdc.), with the latter subspecies containing two varieties (var. gabonica and var. suaveolens). However, population genetic analyses of short plastid DNA sequences revealed that individuals of var. gabonica represent a genetically distinct lineage from var. suaveolens (Dauby et al. 2010). This genetic differentiation between the two varieties was further confirmed based on the analyses of nuclear microsatellites (Piñeiro et al. 2017; Lissambou et al. in prep.). A species delimitation study within *Greenwayodendron*, based on eight nuclear microsatellites (Piñeiro et al. 2016) coupled with a detailed morphometric dataset of 33 vegetative, floral and fruit traits, confirmed the existence of at least four potentially different species within the *G.suaveolens* complex (Lissambou et al. in prep.). Indeed, besides confirming the strong genetic differentiation between the *gabonica* and *suaveolens* varieties, the latter study identified two new taxa. The subspecies usambaricum, although geographically isolated from Central African species, is differentiated from *suaveolens* by vegetative characters, essentially determined by the number of lateral veins (≤ 12 veins in *suaveolens* and ≥ 14 in *usambaricum*. In addition, a molecular phylogeny of the genus based on numerous nuclear markers (unpublished data), confirmed that G.suaveolenssubsp.usambaricum is phylogenetically distinct from G.suaveolenssubsp.suaveolens. Most of these discriminant traits had already been recognised by several authors (Le Thomas 1969; Verdcourt 1969; Dauby et al. 2010; Piñeiro et al. 2016) and argue that the taxon G.suaveolenssubsp.suaveolensvar.gabonica and G.suaveolenssubsp.suaveolensvar.suaveolens can be elevated to the rank of species, as already proposed by Dauby et al. (2010).

These new insights into the genetic and morphological diversity of *Greenwayodendron* spp. warrant the need for a taxonomic revision of the genus. Here, we formally describe two new species and undertake two new combinations.

**Table 1.** Past and currently proposed infrageneric species composition of *Greenwayodendron*.

Previous species delineation	Current species delineation
Greenwayodendron suaveolens subsp. suaveolens var. suaveolens	* Greenwayodendron suaveolens *
Greenwayodendron suaveolens subsp. suaveolens var. gabonica	* Greenwayodendron gabonicum *
Greenwayodendron suaveolens subsp. usambaricum	* Greenwayodendron usambaricum *
* Greenwayodendron oliveri *	* Greenwayodendron oliveri *
NA	* Greenwayodendron littorale *
NA	* Greenwayodendron glabrum *

## Taxonomical history

Oliver (1868) in Flora of Tropical Africa, described Polyalthia?acuminata. Oliver (1868), acknowledging clear morphological differences between the known *Polyalthia* spp. and this new African species, extended the generic diagnosis of *Polyalthia* to accommodate this new entity. However, the species epithet *acuminata* was already occupied by a South Asian species of *Polyalthia* named by Thwaites (1864). Engler and Prantl (1897) erected sect. Afropolyalthia within *Polyalthia*, to accommodate the species described by Oliver (1868) under the new replacement name *P.oliveri* Engl. A few years later, Engler and Diels (1901) published a new species, *P.suaveolens* Engl. & Diels, within the section Afropolyalthia. Subsequently, a number of species were described across Africa: *Polyalthiamortehanii* De Wild (De Wildeman 1914), *Mabagossweileri* Greves (Greves 1929) and *Xylopiaotunga* Exell (Exell 1931) recognised as synonyms of *Greenwayodendronsuaveolens* and *Polyalthiaaubrevillei* Ghesq. ex Aubrév (Aubréville 1936) considered a synonym of *Greenwayodendronoliveri* (Aubréville 1936). Pellegrin (1949) named a new variety from Gabon as Polyalthiaoliverivar.gabonica Pellegrin. This taxon had no Latin diagnosis rendering it as a *nomen nudum*. Le Thomas (1965) however, suggested that this new taxon is a variety of *Polyalthiasuaveolens* (and not *P.oliveri*) and validly published the name Polyalthiasuaveolensvar.gabonica Le Thomas. A few years later, Verdcourt (1969) erected the genus *Greenwayodendron* to accommodate the species described within Polyalthiasect.Afropolyalthia. Indeed, he argued that there were sufficient morphological differences with the Southeast Asian *Polyalthia* species to warrant a new generic status. In addition, he also described Greenwayodendronsuaveolenssubsp.usambaricum Verdc. known from a very small isolated population in Tanzania. The same year, in the Flore du Gabon, Le Thomas (1969) did not accept the new generic name proposed by Verdcourt. Indeed, she postulated that the African species might be closely related to the Asian ones and argued in favour of a more variable *Polyalthia* s.l. circumscription with fewer generic names. However, subsequent morphological (Doyle and Le Thomas 1994; Doyle and Le Thomas 1996) and molecular phylogenetic studies (Chatrou et al. 2012) validated *Greenwayodendron* as a phylogenetically and morphologically distinct genus from *Polyalthia* s.l. Finally, *Polyalthia* s.l. is a mainly Southeast Asian genus (but see these articles for recent changes: Mols et al. 2004; Chatrou et al. 2012; Xue et al. 2014).

## Material and methods

Around 500 specimens of *Greenwayodendron* present in the following herbaria BRLU, BM, BR, COI, K, LBV, P, WAG and YA (abbreviations follows Holmgren et al. (1990)) were examined for this study. The online resources JSTOR Global Plants (http://plants.jstor.org), IPNI (http://www.ipni.org), Tropicos (http://www.tropicos.org), the Museum of Natural History, Paris (http://www.mnhn.fr) and the Herbarium at the University of Coimbra (http://coicatalogue.uc.pt) were consulted for the study of type specimens. Species descriptions are based on living and herbarium specimens. The open source software QGis was used to generate the different taxon distribution maps. The Latin name *Greenwayodendron* is neutral (derived from the Greek word “*dendron*” meaning tree). New epithets names and combinations proposed here are thus concordant with the neutral gender.

Preliminary conservation assessments of each taxon were assessed using the IUCN Red List Categories and Criteria (IUCN 2012) using criterion B based on the distribution of each species inferred from georeferenced herbarium specimens (Schatz 2002). The extent of occurrence (EOO) and the area of occupancy (AOO) were estimated using the *ConR* R package (Dauby et al. 2017). The minimum AOO was estimated based on a user defined grid cell of 4 km^2^, as recommended by IUCN (2017). Each collecting locality was regarded as a separate subpopulation. The number of ‘locations’ (as defined by IUCN 2017) was calculated with regard to the type of threats, such that a single ‘location’ may encompass more than one adjacent subpopulation. Vernacular names are mainly taken from herbarium specimen label information and from Raponda-Walker and Sillans (1961) and Aubréville (1936) for species found in Gabon.

## Results and discussion

### Morphology of *Greenwayodendron*

**Habit.** The different species of *Greenwayodendron* vary from large trees up to 45 metres tall (*G.suaveolens*) to small trees, no higher than 5 m (*G.littorale*). The trunks of *Greenwayodendron* are generally straight and cylindrical with a grey bark covered in large white/grey spots. The slash is bright orange with a distinct black ring under the bark. This black ring is also found in some other African species of Annonaceae such as *Anonidiummannii* Engl. & Diels. In terms of phylotaxis of the main axis, species in *Greenwayodendron* follow the spiral pattern, which is common to all other members of the tribe Piptostigmateae (Johnson 2003).

**Branches.** In terms of pubescence, young branches vary from tomentose (i.e. dense brown pubescence) in *G.gabonicum* to sparsely pubescent to glabrous in the other four species. The pubescence tends to disappear with age and branches are generally sparsely pubescent to glabrous when older. However, in *G.gabonicum*, the pubescence persists even in old branches.

**Leaves.** The leaves of *Greenwayodendron* follow the typical Annonaceae characters: they are estipulate, simple, entire, distichous and alternate. The petiole of all species can be tomentose (*G.gabonicum*) to glabrous (*G.glabrum*). The leaf lamina varies from elliptic to narrowly elliptic in shape, while the base is rounded or cuneate. The apex of the leaves can be very variable even within species. For example, in *G.glabrum*, the apex varies from acuminate to emarginated (see for example Fig. 3D–G). Leaf size varies from 4.2–7.8 cm in *G.littorale* up to 10.0–26.3 cm long in *G.gabonicum*. In most species (*G.glabrum, G.littorale* and *G.oliveri*), the leaf lamina is glabrous on the upper side and sparsely pubescent on the lower side. Only *G.gabonicum* is characterised by a densely pubescent leaf upper side.

**Midrib.** As for most African genera (Couvreur 2009; Le Thomas 1969), the midrib is impressed on the upper side. The midrib varies from densely pubescent on both sides (*G.gabonicum*) to glabrous on both sides (*G.glabrum*). Secondary veins are brochidodromous or loop-forming (in contrast to other common Annonaceae venation type eucamptodromous) with prominent intermarginal veins and secondary veins that are straight then arcuate towards the margin (Klucking 1986; Scharaschkin and Doyle 2006). Tertiary veins are reticulate (forming a network) in all species, in contrast to other genera of the tribe (Couvreur et al. 2015) that are either percurrent (*Brieya*, *Piptostigma*, *Polycertocarpus*) (parallel) or intermediate between reticulate and percurrent (*Annickia* and *Mwasumbia*).

**Inflorescence.** The inflorescences in Annonaceae are characterised by a terminal flower with more lateral branched partial inflorescences (Weberling and Hoppe 1996). The inflorescence of the genus *Greenwayodendron* is terminal (leaf opposed) and generally extra-axillary on old branches. The majority of genera in Piptostigmateae, except *Annickia* Setten & Maas, have axillary inflorescences (Couvreur et al. 2009; Couvreur et al. 2015). Inflorescences are reduced to a compact rhipidium which contains 1–4 flowers. Very often, there is only one flower that develops. In general, the pedicels of all *Greenwayodendron* species are bibracteate, meaning they have two bracts. This conforms to the type 2 of Fries (1955) and is common to most Annonaceae species. The bract, just under the calyx, is referred to as the upper bract and the lower bract corresponds to the bract located in the lower half of the pedicel.

**Flowers.** Flowers in *Greenwayodendron* are actinomorphic, cyclic and trimerous, with 9 tepals. The external whorl of three tepals is typically referred to as sepals, whereas the two inner whorls of three petals each are termed outer and inner petals. *Greenwayodendron* species are androdioecious, with male and hermaphrodite individuals. Within Piptostigmateae, only two other genera, *Sirdavidia* Couvreur & Sauquet and *Polyceratocarpus* Engl. & Diels are also androdiecious, the rest of the genera being bisexual (Couvreur et al. 2009; Couvreur et al. 2015). For *G.glabrum* and *G.littorale*, we did not observe staminate flowers whereas for *G.gabonicum* only male flowers have been seen to date. It is not excluded that the former two species are in fact truly bisexual, but more data needs to be gathered before we can conclude on their sexual systems. Indeed, some African genera of Annonaceae are generally androdioecious but also have functionally bisexual or even monoecious species (Verdcourt 1986; Couvreur and Luke 2010). In contrast to some other African genera such as *Uvariopsis* Engl. (Le Thomas 1969), there is no apparent sexual dimorphism between staminate and hermaphrodite flowers, both being similar in their general aspect such as pedicel length or tepal shape and size. The sepals are small, never longer than 5 mm. The two whorls of petals, are sub-equal in length and similar in shape. We thus did not differentiate them in the descriptions. In vivo, the petals spread out horizontally and recurve slightly downwards towards the apex. Petals range in size from 22–24 mm long in *G.gabonicum* to 11.5–12.5 mm long in *G.littorale*.

In staminate flowers, the stamens range from 16–33 and are packed into several whorls whereas in the bisexual flowers the stamens are fewer in number (4–15) and form a single whorl around the carpels. The connective shield, i.e. the apical extension of the connective between both thecae, is generally tongue shaped and also termed umbonate (Maas et al. 2003) but is flattened and discoid or lobulated in *G.oliveri*. Connective shape has also been shown to be an important taxonomic character in other genera such as *Duguetia* A.St.-Hil. (Maas et al. 2003) or *Uvariastrum* Engl. & Diels (Couvreur 2014). The carpels are free (apocarpic) as for most Annonaceae genera (van Heusden 1992) and vary from 8–20 in number.

**Fruits.** Fruits in *Greenwayodendron* are composed of several shortly stipitate monocarps. The stipes and the fruit pedicels are sparsely pubescent to glabrous and generally the same colour as the monocarp. The monocarps are broadly ellipsoid to globular, sparsely pubescent to glabrous and green to dark purple at maturity. ellipsoid to globular, sparsely pubescent to glabrous and green to dark purple at maturity.

**Seeds.** The seeds of *Greenwayodendron* range from 1–4 per monocarp. They are ellipsoid to globular, flattened when there is more than one seed per monocarp and surrounded by a furrow. The surface of the seed is covered with a rough white integument and the raphe is always impressed.

### Key to the species of *Greenwayodendron*

For a key to the genera of Piptostigmateae, see Ghogue et al. (2017).

**Table:** 

1	Upper side of leaf lamina clearly densely pubescent	*** G. gabonicum ***
–	Upper side of leaf lamina sparsely pubescent to glabrous	**2**
2	Upper side of midrib glabrous	*** G. glabrum ***
–	Upper side of midrib to sparsely pubescent	**3**
3	Connective of the stamens flattened or discoid in shape (West Africa)	*** G. oliveri ***
–	Connective of the stamens tongue-shaped or lobulated (Central and East Africa)	**4**
4	Tree 2–5 m tall, stamens in hermaphrodite flowers 4–5; mature monocarps 2.5 × 4.2 cm in diameter (coastal Gabon and Republic of Congo)	*** G. littorale ***
–	Tree up to 45 m lower limit of height, stamens in hermaphrodite flowers 5–10; mature fruits 7.2 × 16.4 cm in diameter	**5**
5	Secondary veins ≤ 12 (Central Africa, widespread)	*** G. suaveolens ***
–	Secondary veins ≥ 14 (Tanzania, Eastern Arc Mountains)	*** G. usambaricum ***

#### Greenwayodendron

Taxon classificationPlantaeMagnolialesAnnonaceae

Verdc., Adansonia, sér. 2, 9 1. (1969)

Polyalthia sect. Afropolyalthia Engler & Prantl., Leipzig,W. Engelmann.160. (1897)

##### Type species.

*Greenwayodendronoliveri* Engl.

Tree 2–45 m tall, d.b.h. 2–125 cm; stem cylindrical, bark smooth with large white stains, slice orange with a black ring, aromatic and rapidly turning brown. Young branches at first densely pubescent, later glabrous, trichomes 0.1–1.0 mm long; older branches sparsely pubescent to glabrous. Leaves entire, simple, alternate, astipulate; petiole 1.0–11.1 mm long, 0.8–3.3 mm in diameter, densely pubescent to glabrous, trichomes 0.1–1.2 mm long, indument brown; lamina 4.2–26.3 cm long, 2.0–9.6 cm wide, length: width ratio 1.5–4.0; elliptic to narrowly elliptic, base rounded or cuneate, apex acuminate, acute, apiculate, aristate or caudate, acumen 1–40 mm long, upper side sparsely pubescent to glabrous, lower side densely to sparsely pubescent; midrib upper side base densely pubescent to glabrous, lower side densely pubescent to glabrous, trichomes 0.1–1.2 mm long, indument tomentose; secondary veins 4–18 pairs, upper side sparsely pubescent to glabrous, lower side densely pubescent to glabrous, trichomes 0.1–1.0 mm long; tertiary veins upper side sparsely pubescent to glabrous, lower side sparsely pubescent to glabrous, irregularly prominent, slightly raised, distinct or indistinct above. Inflorescence axillary, 1–4 flowered rhipidium. Floral buds ellipsoid, 4–9 mm long, 2–5 mm in diameter, densely covered with short and long trichomes. Flowering pedicel 3.0–6.3 mm long, 0.5–2.2 mm in diameter, densely pubescent when young, becoming glabrous, trichomes 0.1–0.5 mm long; lower bract in lower half of pedicel, minute, sparsely pubescent; upper bract apical, just below the calyx, 0.9–5.2 mm in diameter, sparsely pubescent, trichomes 0.1–0.6 mm long. Sepals 1.3–5.0 mm long, 1.6–4.7 mm wide, length:width ratio 0.5–0.9 broadly ovate, imbricate, fused at the base, apex acuminate, base truncate, densely to sparsely pubescent outside, sparsely pubescent towards the centre inside, trichomes 0.1–0.5 mm long. Inner and outer petals subequal, 8.0–24.6 mm long, 1.3–3.5 mm wide, length:width ratio 0.5–0.9, narrowly ovate to elliptic, apex acuminate, base rounded, green maturing pale yellow, outside tomentose, trichomes 0.1–0.5 mm long, erect, inside sparsely pubescent to glabrous; glabrous part to 2–8 mm long. Male flowers: stamens 10–33, in several whorls, 1.2–4.2 mm long, 0.4–0.9 mm wide, tightly appressed; connective shield of stamens tongue-shaped, lobulated or flattened/discoid; hermaphrodite flowers: stamens 4–10 in a single whorl, appressed, 0.9–2.2 mm long and 0.3–0.8 mm wide, connective stamen tongue-shaped, lobulated or flattened; carpels 8–20, 0.7–2.1 mm long, 0.5–0.9 mm in diameter, length:width ratio 1.2–2.8 narrowly oblong, densely pubescent; ovules 1–2, oblong; stigmata ovoid, densely pubescent, trichomes 0.1–0.5 mm long. Fruiting pedicel 4.5–13.1 mm long, 1.0–3.5 mm in diameter, sparsely pubescent to glabrous, trichomes ca. 0.1–0.5 mm long; stipes 3.0–12 mm long and 1.0–3.2 mm in diameter, sparsely pubescent; monocarps 1–8, 2.5–21.1 mm in diameter, broadly ellipsoid to globose, sparsely pubescent to glabrous, green turning wine red at maturity; seeds 1–4 per monocarp, 3.0–15.4 mm in diameter, ellipsoid to globose, flattened on one side or hemi-ellipsoid when more than one seed per monocarp, surface covered by a white tegument; raphe impressed.

#### Greenwayodendron gabonicum

Taxon classificationPlantaeMagnolialesAnnonaceae

(Le Thomas) Lissambou & Couvreur comb. nov.

urn:lsid:ipni.org:names:77192855-1

Fig. 1

Polyalthia suaveolens (Engl. & Diels) var. gabonica Pellegr. ex Le Thomas., Muséum National d’Histoire Naturelle. Paris. Vol. 16. 206. (1969) ≡ Greenwayodendronsuaveolensvar.gabonica (Pellegr., ex Le Thomas) Verdc., Adansonia, sér. 2, 9 (1). (1969).

##### Type.

**GABON.** Ogooué-Lolo: Région de Lastoursville, 25 Feb 1930, *G.M.P.C. Le Testu 7936* (lectotype, designated by Le Thomas 1965 p. 453, P![P00363322]; isotypes: BM web [BM000547163]; BR![BR880441]; P![P0036331]).

Tree 4–20 m tall, d.b.h. 4–40 cm in diameter. Young branches densely to sparsely pubescent, trichomes 0.5–1.0 mm long; old branches sparsely pubescent. Leaves: petiole 4.0–11.1 mm long, 1.0–3.3 mm in diameter, densely to sparsely pubescent, trichomes 0.4–1.2 mm long, indumenta brown; lamina 10.0–26.3 cm long, 4.0–9.6 cm wide, length:width ratio 1.9–3.6, elliptic to narrowly elliptic, base rounded cuneate, apex acuminate, acute, apiculate or emarginate, acumen 8–35 mm long, upper side densely pubescent, lower side densely pubescent; midrib upper and lower sides densely pubescent, trichomes 0.5–1.2 mm long, indumenta tomentose; secondary veins 6–11 pairs, upper side pubescent, lower side densely pubescent, trichomes 0.3–1.0 mm long; tertiary veins upper side sparsely pubescent, lower side densely pubescent, irregularly prominent or indistinct above. Inflorescence axillary, a 1–4 flowered rhipidium. Floral buds ellipsoid, 6–9 mm long, 2.0–3.2 mm in diameter, densely covered with long trichomes. Flowering pedicel 4.5–6.0 mm long, 2.0–2.2 mm in diameter, densely pubescent, trichomes ca. 0.5 mm long, lower bract in lower haft of pedicel, minute, upper bract apical, just below the calyx, 4.8–5.2 mm in diameter, densely pubescent, trichomes ca. 0.6 mm long. Sepals 3.5–4.1 mm long, 4.5–4.7 mm wide, length:width ratio 0.8–0.9 broadly ovate, imbricate, fused at the base, apex acuminate, base truncate, outside pubescent, inside sparsely pubescent, trichomes 0.1–0.5 mm long. Inner and outer petals 22.7–24.6 mm long, 2.3–3.5 mm wide, length:width ratio 0.8–0.9, narrowly ovate to narrowly elliptic, apex acuminate, base rounded; outside tomentose, trichomes 0.5–0.6 mm long; inside sparsely pubescent to glabrous; glabrous part to 4.2–4.6 mm long from the base; petals green maturing pale yellow. Male flowers: stamens 24–33, in several whorls, 3.2–4.2 mm long, 0.4–0.6 mm wide, tightly appressed; connectives tongue-shaped or lobulated. Hermaphrodite flowers: not observed. Fruiting pedicel 7.0–13.1 mm long, 2.0–3.5 mm in diameter, sparsely pubescent, trichomes ca. 0.5 mm long; stipes 5.5–12.0 mm long, 1.5–3.1 mm in diameter, sparsely pubescent; monocarps 1–8, 11.0–19.5 mm in diameter, broadly ellipsoid to globose, sparsely pubescent to glabrous, green turning wine red at maturity; seeds 1–4 per monocarp, 7.8–15.4 mm in diameter, ellipsoid to globose, hemispherical or flattened, flattened on one side when more than one seed per monocarp, surface covered by a white tegument.

**Figure 1.**
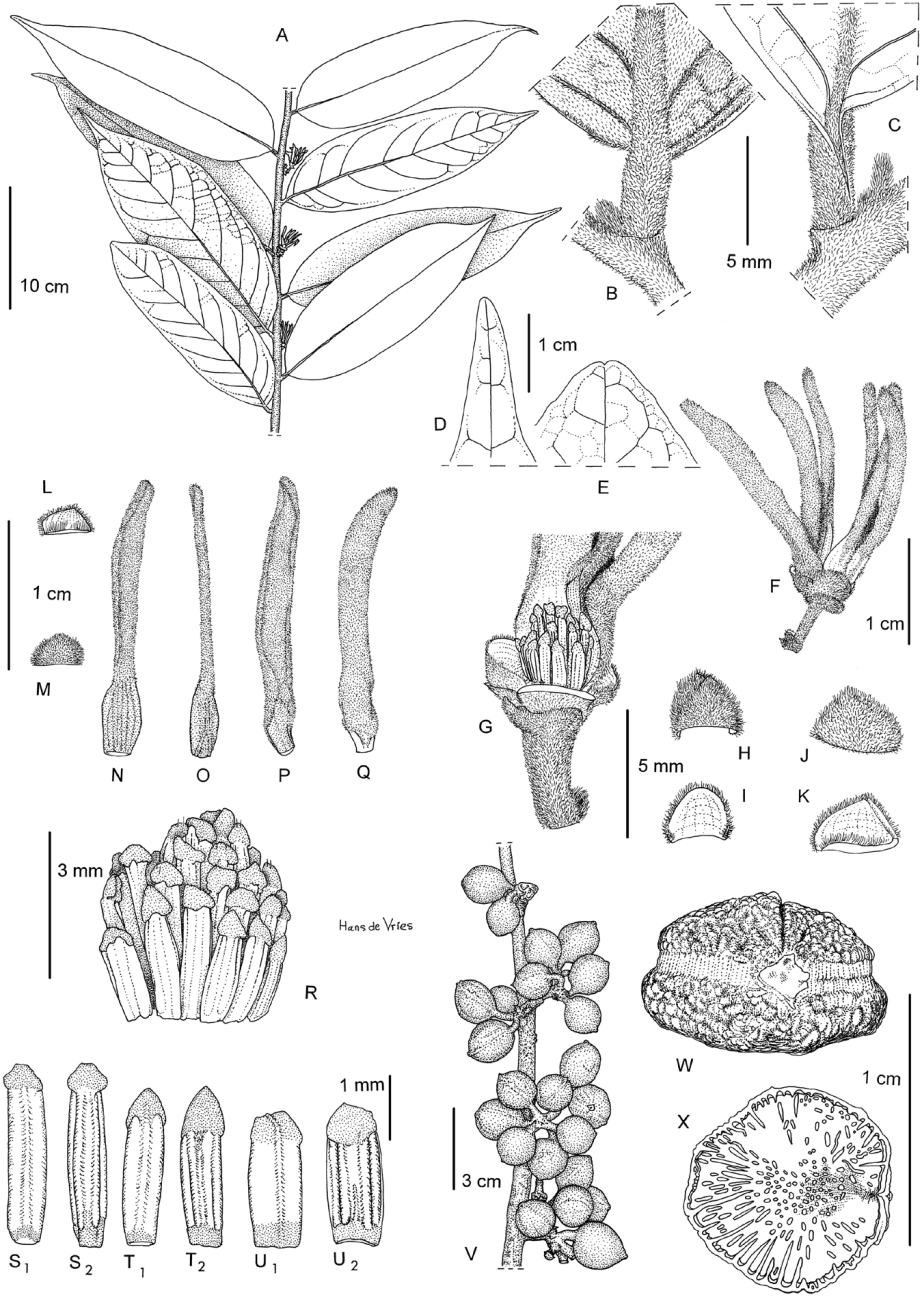
*Greenwayodendrongabonicum*. **A** Flowering branch **B** Detail of lower leaf surface **C** Detail of upper leaf surface **D-E** Detail of leaf apex **F** Flower **G** Detail of male receptacle, inner and outer petals removed **H** Outside view of basal bract **I** Inside view of basal bract **J** Outside view of upper bract **K** Inside view of upper bract **L** Inside view of sepal **M** Outside view of sepal **N** Inside view of outer petal **O** Outside view of outer petal **P** Outside view of inner petal **Q** Inside view of inner petal **R** Detail of androecium **S** Detail of inner row of anthers (**S1** Inside view, **S2** Outside view) **T** Detail of outer row of anthers (**T1** Inside view, **T2** Outside view) **U** Detail of outer row of anthers, different morphology **(U1** Inside view, **U2** Outside view) **V** Fruiting branch **W** Seed, latitudinal view **X** Longitudinal section of seed showing ruminations. **A***Le Testu, G.M.P.C. 7936***B–U2***McPherson, G. 13736***V–X***McPherson, G. 15498*. Drawing by Hans de Vries.

##### Distribution.

**Figure 2.**
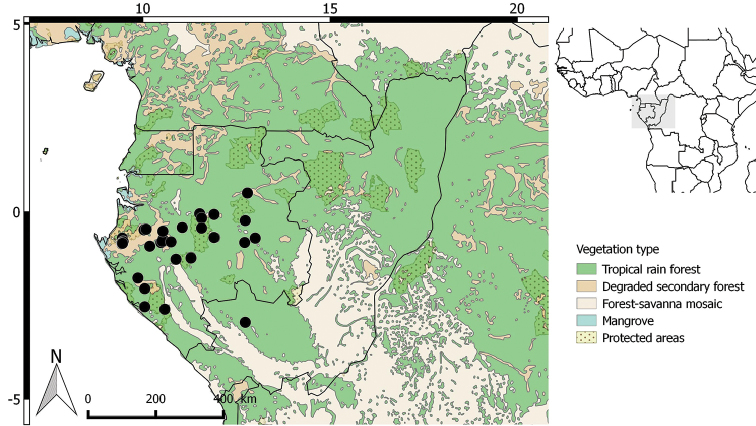
Distribution of *Greenwayodendrongabonicum*.

Mainly occurring in Gabon and one collection from the Republic of Congo; 10–500 m (Fig. 2).

##### Habitat and ecology.

In primary and secondary forests, also occurring in forest-savannah mosaics (Lope and Wonga Wongué).

##### Phenology.

In Gabon, *G.gabonicum* flowers from January to March, immature fruits May to October and mature fruits November to December (Amman Bush; Personal communication), also based on herbaria.

##### Vernacular names.

Gabon: Mutunga (Aduma, Awadji, Nzebi), Otunga (Fang, Kota, Obamba).

##### Preliminary conservation status of IUCN.

Least Concern [LC]. The extent of occurrence (EOO) of *Greenwayodendrongabonicum* is estimated to be over 106,375.19 km^2^, whereas its area of occupancy (AOO) is estimated to be 128 km^2^ (which falls within the limits for Vulnerable status under criterion B2). The species, recorded from Gabon and the Republic of Congo, is now known from at least 35 specimens representing 22 subpopulations. These 22 subpopulations represent 20 different locations (sensu IUCN 2012), many more than 10 locations, which are the upper limit for Vulnerable status under the subcriterion ‘a’. *Greenwayodendrongabonicum* has been collected in 5 protected areas in Gabon (National Park: Moukalaba-Doudou, Lopé, Ivindo, Waka) and Wonga Wongue Reserve and from unprotected areas. This taxon is relatively low in abundance except in two localities (Wonga Wongue Reserve and Lopé Park) where the relative abundance is high. The main threat to *G.gabonicum* is its habitat destruction resulting from logging activities, especially in the coastal part of Gabon. Notwithstanding these human activities, with varying levels of impact, the species appears not as threatened as it is an abundant and quite widespread species. The available information suggests that the number of subpopulations and mature individuals of *G.gabonicum*, as well as its EOO and AOO, will not decrease noticeably in the near future.

##### Uses.

Unknown.

##### Notes.

*Greenwayodendrongabonicum* is easily differentiated from all other species of the genus by its dense tomentose pubescence mainly along the petiole and midrib. In addition, *G.gabonicum* is the only species to have a densely pubescent or sparsely pubescent upper leaf lamina and has the longest leaves, petals and monocarps of the genus.

This species was initially described as a variety of *G.suaveolens*. However, phylogeographics (Dauby et al. 2010), genetic data (Piñeiro et al. 2017; Lissambou et al. in prep.) and morphological characters (leaf size, pubescent, flower and fruit size) clearly support the hypothesis that this entity represents a distinct species altogether leading us to make this new combination.

Since there were two specimens of *Le Testu 7936* in Paris, we chose the specimen barcoded P00363322 as the holotype of *G.gabonicum*. Specimen P0036331 is thus an isotype.

This species was, until recently, suggested as a strict endemic to Gabon, however it has also been collected in the Republic of Congo (*P. Sita 4045*) in the Niari region (Fig. 2).

##### Selected specimens examined.

**Gabon. Haut-Ogooué**: Ossélé village, 45 km on road from Franceville to Kessala, 1°51.28'S, 13°50.80'E, 23 Mar 2015, *Couvreur, T.P.L. 746* (WAG, LBV, BRLU). **Moyen-Ogooué**: Zone de Mabounié, 45 km southwest of Lambaréné, north bank of the Ngounié River, 0°26.706'S, 10°19.458'E, 13 Dec 2012, *Bidault, E. 800* (BRLU, LBV, MO); Concession Rougier du Haut-Abanga, southeast of Mikongo, northern part of the Mekie Mountains, 0°24.135'N, 11°13.212'E, 13 Jun 2008 *Dauby, G. 909* (BRLU, LBV, MO); *ibid. loc.*, 0°46.3956'S, 10°28.3332'E, 10 May 2012, *Dauby, G. 2809* (BRLU, LBV, MO). **Ngounié**: *ibid. loc.*, 0°28.854'S, 10°18.846'E, 14 Nov 2013, *Bidault, E. 1297* (BRLU, LBV, MO); *ibid. loc.*, 0°47.729'S, 10°31.991'E, 8 May 2012, *IRD plot 91* (BRLU, LBV, MO). **Nyanga**: Chantier CEB, ca. 35 km SW of Doussala, 2°30'S, 10°30'E, 18 May 1985, *Reitsma, J.M. 1030* (LBV, WAG); Inventory, chantier CEB, ca. 50 km SW of Doussala, 2°36'S, 10°35'E, 19 Oct 1985, *Reitsma, J.M. 1679* (LBV). **Ogooué Ivindo**: Route chantier Doti 3 – Leroy Gabon. Forêt des abeilles, 0°41'S, 11°54'E, 5 Oct 1993, *Gesnot 8* (BRLU, LBV, MO); Forêt des abeilles, 0°41'S, 11°54'E, 15 Nov 1993, *Gesnot 165* (BRLU, LBV, MO); Station de Recherche de l’Institut de Recherche en Ecologie tropicale (IRET-Ipassa), 0°30.303'N, 12°47.748'E, 18 Dec 2014, *Lissambou, B.J. 300* (BRLU, LBV); *ibid. loc.*, 0°28.62'N, 12°46.71'E, 28 Apr 2015, *Lissambou, B.J. 1134* (BRLU, LBV); Réserve de la Lopé au sud d’Ayem, chantier Soforga, 0°25'S, 11°30'E, 5 Mar 1989, *McPherson, G.D. 13716* (LBV); South of Ayem, western border of Lopé-Okanda Reserve, 0°25'S, 11°30'E, 9 Nov 1991, *McPherson, G.D. 15498* (BR, LBV, P, WAG). **Ogooué Lolo**: Région de Lastoursville, 0°49'S, 12°43'E, 25 Feb 1930, *Le Testu, G.M.P.C. 7936* (BR, P). **Ogooué Maritime**: Région du lac Alombié, +/- 10 km to the north of Mpaga, 0°29.412'S, 9°16.3608'E, Oct 2014, *Lachenaud, O.L.S. 1926* (LBV, BRLU); Mpaga. Département de Bendjé, 0°50.005'S, 9°27.771'E, 11 Oct 2014, *Lissambou, B.J. 0001* (BR, BRLU, L, LBV, MO, P); *ibid. loc.*, 0°50.030'S, 9°26.175'E, 11 Oct 2014, *Lissambou, B.J. 0004* (BR, BRLU, L, LBV, MO, P); Mpaga. Département de Bendjé, SW of Lambaréné, near Lake Ezanga; Conoco drilling site; sandy soil, 1°0.345'S, 10°11.818'E, 13 Feb 1991, *McPherson, G.D. 15293* (LBV).

**The Republic Congo**. **Niari**: Chaillu, Région de Komono, about Mbaya Mossendjo road, 2°57'S, 12°43'E, 15 Nov 1976, *Sita, P. 4045* (BR).

#### Greenwayodendron glabrum

Taxon classificationPlantaeMagnolialesAnnonaceae

Lissambou, Hardy & Couvreur sp. nov.

urn:lsid:ipni.org:names:77192856-1

Fig. 3

##### Diagnosis.

*Greenwayodendronglabrum* is morphologically similar to *G.suaveolens* but differs from it by the absence of trichomes on the petiole, midrib and lower and upper side of the leaf lamina.

**Figure 3.**
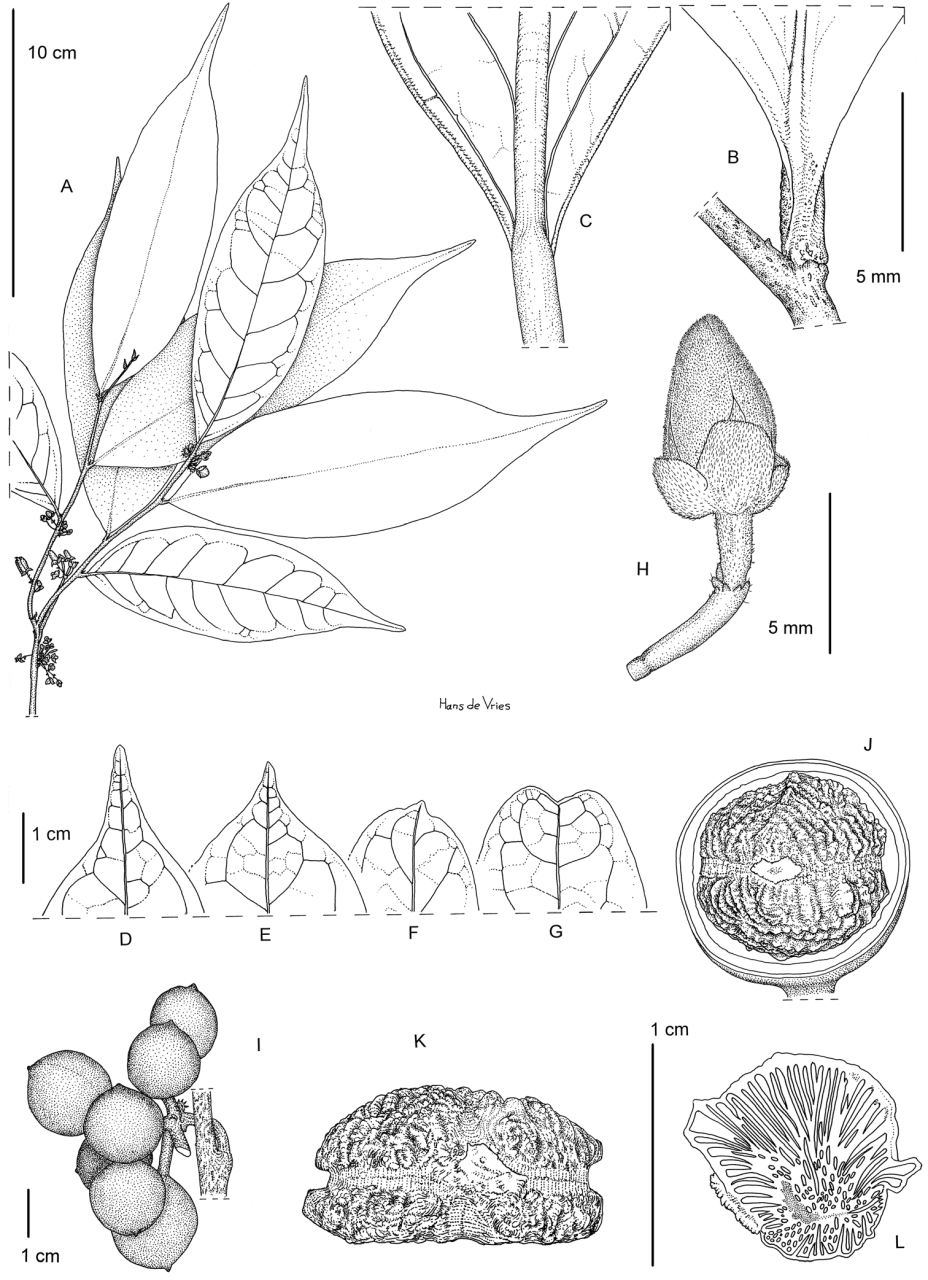
*Greenwayodendronglabrum*. **A** Flowering branch **B** Detail of lower leaf surface **C** Detail of upper leaf surface **D–G** Different types leaf apex **H** Flower bud **I** Infructescence **J** Longitudinal section of fruit revealing seed **K** Seed, latitudinal view **L** Longitudinal section of seed showing ruminations. **A–C, G–H***Letouzey, R. 12869***D–F, I–L** Bos, J.J. 6267. Drawing by Hans de Vries.

##### Type.

**CAMEROON. South region**: 40 km from Kribi, 5 km E of Edea road, tract of Fifinda-Bella road (SFIA), 6 Feb 1970, *J.J. Bos* 6267 (holotype WAG! [WAG1433854]; isotypes (WAG! [WAG1433855]; YA)

Tree 7–30 m tall, d.b.h. 3–20 cm. Young branches sparsely pubescent trichomes ca. 0.1 mm long; old branches glabrous. Leaves: petiole 2.5–6.2 mm long, 1.0–2.2 mm in diameter, glabrous; lamina 6.5–16.2 cm long, 2.1–5.8 cm wide, length: width ratio 2.2–3.6; elliptic to narrowly elliptic, base rounded or cuneate, apex acuminate, aristate, apiculate or caudate, acumen 4–40 mm long, upper side glabrous, lower side sparsely pubescent to glabrous; midrib glabrous on the upper and lower side; secondary veins 5–7 pairs, upper side glabrous, lower side sparsely pubescent to glabrous, trichomes ca. 0.1 mm long; tertiary veins irregularly and indistinct above. Inflorescence axillary, a 1–4 flowered rhipidium. Floral buds ellipsoid, 4.0–4.5 mm long, 3.5–4.0 mm in diameter, densely covered with short trichomes. Flowering pedicel 3.5–4.0 mm long, 0.5–1.1 mm in diameter, sparsely pubescent covered with short trichomes, becoming glabrous, trichomes 0.1 mm long, lower bract in lower haft of pedicel, minute, densely pubescent, upper bract apical, just below the calyx, 2.0–2.2 mm in diameter, densely pubescent, trichomes 0.2–0.3 mm long. Sepals 3.0–3.1 mm long, 3.2–3.5 mm wide, length:width ratio ca. 0.9, broadly ovate, imbricate, fused at the base, apex acuminate, base truncate, densely pubescent outside, sparsely pubescent inside, trichomes 0.1–0.2 mm long. Inner and outer petals 12–13 mm long, 2.0–2.5 mm wide, length:width ratio ca. 0.9, narrowly ovate to narrowly elliptic, apex acuminate, base rounded; outside tomentose, trichomes ca. 0.3 mm long; inside sparsely pubescent to glabrous; glabrous part to 4.2–4.6 mm long; green maturing pale yellow. Male flowers: not observed. Hermaphrodite flowers: stamens 10–15 in a single whorl, appressed, 1.2–1.5 mm long and 0.3–0.4 mm wide, stamens tongue-shaped or lobulated in shape; carpels 10–15, 1.1–1.2 mm long, 0.5–0.6 mm in diameter, length:width ratio 2.8, narrowly oblong, densely pubescent; ovules 1–2, oblong; stigmata ovoid, densely pubescent, trichomes ca. 0.1–0.2 mm long. Fruiting pedicel 6–13 mm long, 1.3–2.2 mm in diameter, sparsely pubescent, trichomes ca. 0.2 mm long; stipes 4.5–10.2 mm long and 1.1–3.2 mm in diameter, glabrous; monocarps 2–8, 11.0–21.1 mm in diameter, broadly ellipsoid to globose, sparsely pubescent to glabrous, green turning wine red at maturity; seeds 1–4 per monocarp, 7.0–12.6 mm in diameter, ellipsoid to globose, flattened on one side or hemi-ellipsoid when more than one seed per monocarp, surface covered by a white tegument.

##### Distribution.

**Figure 4.**
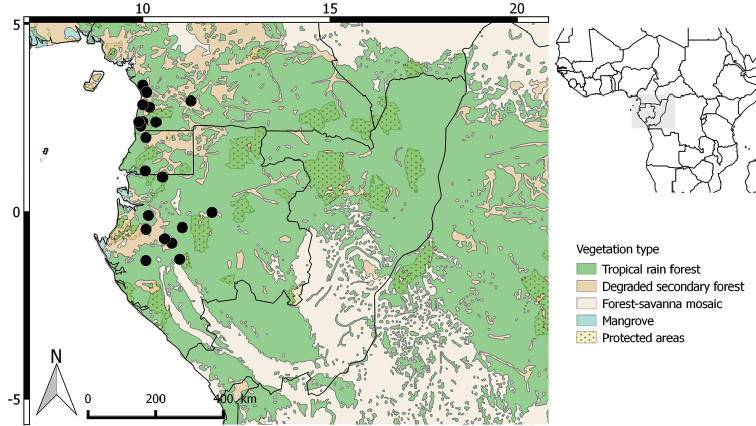
Distribution of *Greenwayodendronglabrum*.

Distributed in south-western Cameroon and north-western and central Gabon; 20–750 m (Fig. 4).

##### Habitat and ecology.

A sub-canopy tree, in dense moist evergreen mature and secondary forests.

##### Phenology.

Flowering and fruiting are not well known. A flowering sample was collected in February and several fruiting samples were collected in November in Gabon and in February in Cameroon.

##### Vernacular names.

Unknown.

##### Uses.

Unknown.

##### Preliminary conservation status of IUCN.

Least Concern [LC]. The extent of occurrence (EOO) of *Greenwayodendronglabrum* is estimated to be over 78,284.28 km^2^, whereas its area of occupancy (AOO) is estimated to be 108 km^2^ (which falls within the limits for Vulnerable status under criterion B2. The species, recorded from Gabon and Cameroon, is now known from at least 31 specimens representing 22 subpopulations. These 20 subpopulations represent 20 different locations (sensu IUCN 2012), many more than 10 locations, which are the upper limit for Vulnerable status under subcriterion ‘a’. *Greenwayodendronglabrum* has been collected in 3 protected areas in Cameroon (Campo-Ma’an National Park), Equatorial Guinea (Monte Alen Nation), Gabon (National Park of Crystal Mountains) and from unprotected areas. This taxon has a low relative abundance except in localities from the region going from the northwest of Gabon to the south of Cameroon where the relative abundance is high. The main threat to *G.glabrum* is its habitat destruction resulting from logging activities and intensive agriculture in Cameroon. Notwithstanding these human activities, with varying levels of impact, the species appears not as threatened as it is abundant. The available information suggests that the number of subpopulations and mature individuals of *G.glabrum*, as well as its EOO and AOO, will not decrease noticeably in the next ten years.

##### Notes.

This species was found growing in sympatry with *G.suaveolens* in southern Cameroon. However, *G.glabrum* differs from other species of the genus by the absence of trichomes on the petioles, midrib and lower and upper side of the leaf lamina. Nevertheless, some specimens do present small isolated tufts of trichomes on the lower side of the leaf lamina. This general lack of pubescence was observed both *in situ* on fresh specimens (adults and juveniles) but also on herbarium samples. This species is also genetically distinct from *G.suaveolens* and other species in the genus based on DNA microsatellite data (Lissambou et al. in prep.) and a nuclear gene phylogenetic analysis (Couvreur et al. in prep).

##### Selected specimens examined.

**Cameroon. South Province**: 40 km from Kribi, 5 km E of Edea road, tract of Fifinda-Bella road (SFIA), 3°13'N, 10°04'E, 6 Feb 1970, *Bos, J.J. 6267* (BR, P, WAG, YA,); Mvindi 35 km E of Campo, 2°24'N, 10°21'E, 19 Dec 1983, *Kaji, M. 4* (YA, P); Left bank Nyong R., 30 km S of Edea, near [a/the] bridge on [a/the] road to Kribi, 3°33'N, 9°59'E, *Leeuwenberg, A.J.M. 5582* (BR); Parc Campo MA’AN, 2°17.11572'N, 9°57.006'E, 17 Jan 2016, *Lissambou, B.J. 1745* (BRLU); *ibid. loc.*, 2°17.110'N, 9°56.812'E, 17 Jan 2016, *Lissambou, B.J. 1755* (BRLU); *ibid. loc.*, 2°17.5189'N, 9°56.730'E, 18 Jan 2016, *Lissambou, B.J. 1807* (BRLU); Concession forestière Wishema, 2°24.446'N, 9°53.781'E, 19 Jan 2016, *Lissambou, B.J. 1828* (BRLU); *ibid. loc.*, 2°24.411'N, 9°53.698'E, 19 Jan 2016, *Lissambou, B.J. 1830* (BRLU). **Littoral**: Near Ndogtima Nyong (15 km NE from mouthpiece Nyong) Edea, 3°23'N, 10°00'E, 3 Feb 1974, *Letouzey, R. 12869* (BR, YA, P); Canton du Ntem, 16 km SW from Nyabessan, 2°48'N, 10°11'E, 30 Nov 1982, *Nkongmeneck, B.A. 400* (YA, P).

**Gabon. Estuaire**: Tchimbele, 0°37.07'N, 10°23.57'E, 14 Feb 2010, *Phillippe 83* (BRLU, LBV, MO). **Moyen Ogooué**: Lac Azingo. Grands lacs Moyen Ogooué, 0°28.66'S, 10°1.98'E, 16 Oct 2014, *Lissambou, B.J. 0013* (BR, BRLU, L, LBV, MO, P); *ibid. loc.*, 0°27.768'S, 10°5.2044'E, 17 Oct 2014, *Lissambou, B.J.0014* (BR, BRLU, L, LBV, MO, P); *ibid. loc.*, Lac Azingo. Grands lacs, 0°27.773'S, 10°5.238'E, 26 Oct 2014, *Lissambou, B.J. 0015* (BR, BRLU, L, LBV, MO, P). **Ngounié**: Mabounié, forest on the south side of the N’gounié River, 0°43.116'S, 10°35.933'E, 15 Oct 2012, *Bidault, E. 847* (BRLU, LBV, MO); Upper Waka area, ca.3 km road Mikanda Forestry Camp to Ekanga, 1°18'S, 10°50'E, 29 Mar 2004, *Wieringa, J.J. 5129* (WAG). **Ogooué Ivindo**: South of Ayem; western border of Lopé-Okanda Reserve, 0°25'S, 11°30'E, 19 May 1992, *McPherson, G.D. 15802* (BR, P).

**Equatorial Guinea. Rio Muni, Centro-Sur**: SW from Monte Alén National Park, on the Mosumo Ecofac transect at 500 m from the beginning of the ride, 1°30'N, 10°04'E, 10 Feb 2001, *Senterre, B. 171* (BRLU, LBV, MO); SW from Monte Alén National Park, 200 m S of Transcito Ecofac de Mosumo at 1620 m from the start of the trail, 1°35'N, 10°03'E, 7 Mar 2001, *Senterre, B. 697* (BRLU, LBV, MO).

#### Greenwayodendron littorale

Taxon classificationPlantaeMagnolialesAnnonaceae

Lissambou, Dauby & Couvreur sp. nov.

urn:lsid:ipni.org:names:77192857-1

Fig. 5

##### Diagnosis.

*Greenwayodendronlittorale* resembles *G.oliveri* by being small trees and from the shape and size of their leaves. *Greenwayodendronlittorale* is however different, being smaller in size (2–5 m versus 5–10 m for *G.oliveri*) and the shape of the stamen connectives being tongue-shaped, obtuse or short versus flattened in *G.oliveri*.

##### Type.

**GABON.** Ogooué-Maritime: Gamba, ca. 2 km on sand-track to Sete Cama, 15 Mar 1994, *J.J. Wieringa 2476* (holotype: WAG! [WAG0065156]; Isotypes (BM, BR![BR0000015305985], C, EA, FHO, IAGB, IEC, K![K001595], LBV!, LY, MA, MO, MPU, P![P06900984], PRE, UGDA, U![U0045159; U0045160], US, W, WAG! [WAG0065155; WAG0065154], YA, Z.)

**Figure 5.**
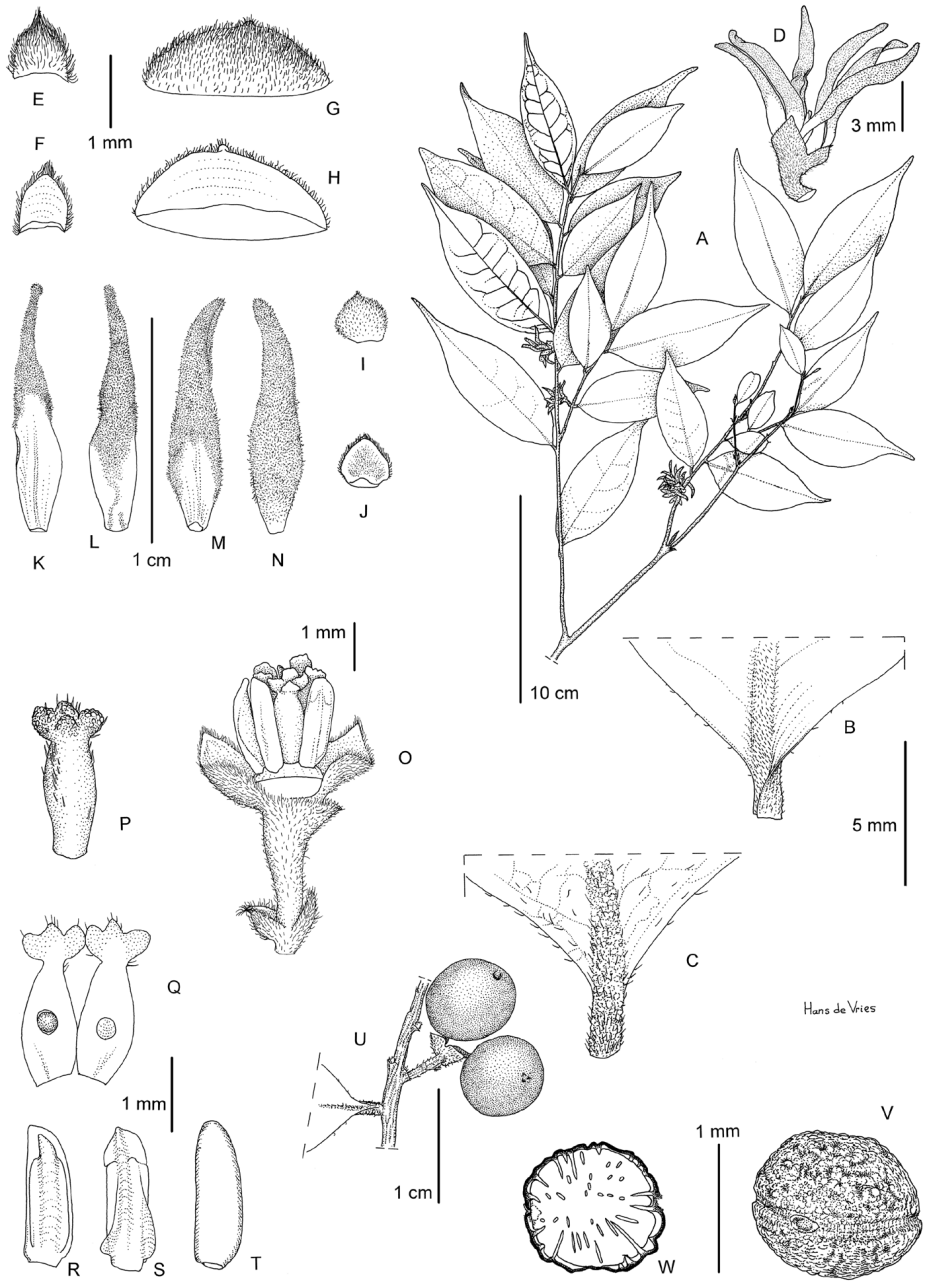
*Greenwayodendronlittorale*. **A** Flowering branch **B** Detail of lower leaf surface **C** Detail of upper leaf surface **D** Flower **E** Outside view of basal bract **F** Inside view of basal bract **G** Outside view of upper bract **H** Inside view of upper bract **I** Inside view of sepal **J** Outside view of sepal **K** Inside view of outer petal **L** Outside view of outer petal **M** Outside view of inner petal **N** Inside view of inner petal **O** Detail of hermaphrodite receptacle showing stamens and carpels, all petals removed **P** Detail of one carpel **Q** Longitudinal section of two carpels, showing single ovule **R** Detail of stamen, outside view **S** Detail of stamen, inside view **T** Detail of stamen, inside view **U** Fruiting branch **V** Seed, latitudinal view **W** Longitudinal section of seed showing ruminations **A-T***Wieringa, J.J. 2476***U–W***Breteler, F.J. 5649*. Drawing by Hans de Vries.

Tree 2–5 m tall, d.b.h. 2–5 cm. Young branches at first sparsely pubescent, later glabrous, trichomes ca. 0.1 mm long; old branches glabrous. Leaves: petiole 1.0–3.1 mm long, 0.8–1.2 mm in diameter, sparsely pubescent, trichomes ca. 0.1 mm long; lamina 4.2–7.8 cm long, 2.0–3.8 cm wide, length: width ratio 1.5–2.3, elliptic, base rounded or cuneate, apex acuminate, aristate or caudate, acumen 5–12 mm long, upper and lower side sparsely pubescent; midrib upper side and lower side densely to sparsely pubescent, trichomes ca. 0.1 mm long; secondary veins 4–6 pairs, upper side glabrous, lower side sparsely pubescent, trichomes 0.1 mm long; tertiary veins irregular, indistinct above. Inflorescence axillary, a 1–4 flowered rhipidium. Floral buds ellipsoid, 5.0–5.5 mm long, 2.5–3.0 mm in diameter, densely pubescent; Flowering pedicel 3.0–3.1 mm long, 0.5–0.6 mm in diameter, densely pubescent when young, becoming pubescent to sparsely pubescent at anthesis, trichomes 0.1 mm long, lower bract in lower haft of pedicel, minute; upper bract apical, just below the calyx, 0.9–1.0 mm in diameter, sparsely pubescent, trichomes 0.2–0.3 mm long. Sepals 1.5–1.9 mm long, 1.9–2.2 mm wide, length: width ratio 0.9 broadly ovate, imbricate, fused at the base, apex acuminate, base truncate, outside densely pubescent, inside sparsely pubescent towards the centre inside, trichomes 0.1–0.2 mm long. Inner and outer petals 11.5–12.5 mm long, 1.6–1.8 mm wide, length: width ratio 0.9, narrowly ovate to narrowly elliptic, apex acuminate, base rounded, outside tomentose, trichomes 0.1–0.2 mm long, inside sparsely pubescent to glabrous, glabrous part to 0.5–1.2 mm long, green maturing pale yellow. Male flowers not observed. Hermaphrodite flowers: stamens 4–5 in a single whorl, appressed, 1.7–2.1 mm long, 0.6–0.9 mm wide, connective tongue-shaped, obtuse, short and little developed; carpels 8–10, 1.9–2.1 mm long, 0.6–0.8 mm wide, length:width ratio 2.8, oblong, densely pubescent; ovules 1, oblong; stigmata ovoid, densely pubescent, trichomes ca. 0.1 mm long. Fruiting pedicel 4.5–6.5 mm long, 1.5–2.0 mm in diameter, sparsely pubescent, trichomes ca. 0.1 mm long; stipes 3–4 mm long and 1–2 mm in diameter, sparsely pubescent; monocarps 1–4, 2.5–4.2 mm in diameter, broadly ellipsoid to globose, sparsely pubescent to glabrous, green turning wine red at maturity; seed 1 per monocarp, 1.5–4.0 mm in diameter, ellipsoid to globose, surface covered by a white tegument.

##### Phenology.

Flowering and fruiting times are not well known. However, a flowering specimen was collected in March and a fruiting one in September.

##### Distribution.

**Figure 6.**
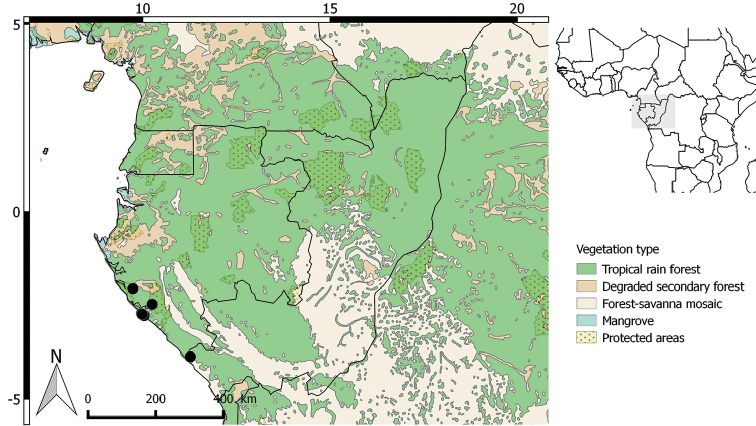
Distribution of *Greenwayodendronlittorale*.

Restricted to the southern coastal part of Gabon and northern Republic of Congo, 5–50 m (Fig. 6).

##### Habitat and ecology.

Growing on coastal and periodically inundated forests, on sandy soils.

##### Vernacular names.

Unknown.

##### Use.

Unknown.

##### Preliminary conservation status of IUCN.

Endangered [EN]. The extent of occurrence (EOO) of *Greenwayodendronlittorale* is estimated to be over 4,506 km^2^ and its minimal area of occupancy (AOO) is estimated to be 24 km^2^ (within the limits for Endangered status under criterion B2). *Greenwayodendronlittorale* is endemic to western Gabon and the Republic of Congo and develops in the lowland coastal forest where it is a dominant species in the undergrowth. The species is found in protected areas (Loango National Park). It is also known from several unprotected forests subjected to logging and habitat destruction due to human activities. *Greenwayodendronlittorale* is known from eight specimens representing five subpopulations. These 5 subpopulations represent a total of 5 “locations” (*sensu*IUCN 2012), falling within the limit for Endangered status. We project that the ongoing loss of its habitat will induce a continuous decline in the number of mature individuals. *Greenwayodendronlittorale* is therefore assigned a preliminary status of EN B1 ab(iii,v)+2ab(iii,v).

##### Notes.

*Greenwayodendronlittorale* was previously confused with *G.oliveri* from West Africa. Besides their clear allopatric distribution (West versus Central Africa), both species are distinct at the morphological level with *G.oliveri* being a taller tree (5–10 m versus 2–5 m) with usually longer leaves (up to 15 cm long versus up to 8 cm long). Both species also differ in the shape of the stamen connective, being flattened in *G.oliveri* and tongue-shaped, obtuse, short and little developed in *G.littorale*. Finally, genetic studies also confirm the distinct nature of both species (Lissambou et al. in prep.). To date, only hermaphrodite flowers were observed.

##### Selected specimens examined.

**Gabon. Nyanga**: 6 km southeast of Mayumba, 3°28.21'S, 10°41.90'E, 20 Nov 2015, *Wieringa, J.J. 8490* (LBV, WAG). **Ogooué Maritime**: Gamba, 2°46'S, 10°20'E, 26 Sep 1968, *Breteler, F.J. 5649* (LBV, WAG); Near Nyanga river, S of Gamba, 2°28'S, 10°15'E, 25 Jul 1998, *Breteler, F.J. 14481* (WAG); Setté Cama, 2°32'S, 9°46'E, 23 Apr 1997, *McPherson, G.D. 16812* (LBV); Gamba. 9.1 km N of Gamba-airport along production road branching from road to Ndogo wharf, 2°44.70'S, 9°59.70'E, 28 Dec 1994, *Wild, J.J.F.E. de 11217* (LBV, BRLU).

**Republic of Congo. Kouilou**: P.C.A. NZAMBI, around N’tiétié, N’Gongo forest road 4 km from N’Tiété, 3°52'S, 11°16'E, 29 Apr 1974, *Sita, P. 3698* (P).

#### Greenwayodendron oliveri

Taxon classificationPlantaeMagnolialesAnnonaceae

(Engl) Verdc., Adansonia sér. 2, 9: 92. (1969).

Fig. 7

Polyalthia oliveri Engl. & Prantl., Leipzig,W. Engelmann.160. (1897).

##### Type.

**IVORY COAST. Lagunes**: Bagroo River, 1961, *G.O. Mann* 841 (lectotype, designated by Verdcourt 1969 p. 92, K![K000580895]; isolectotypes K![K000580894], P![P00363358]).

**Figure 7.**
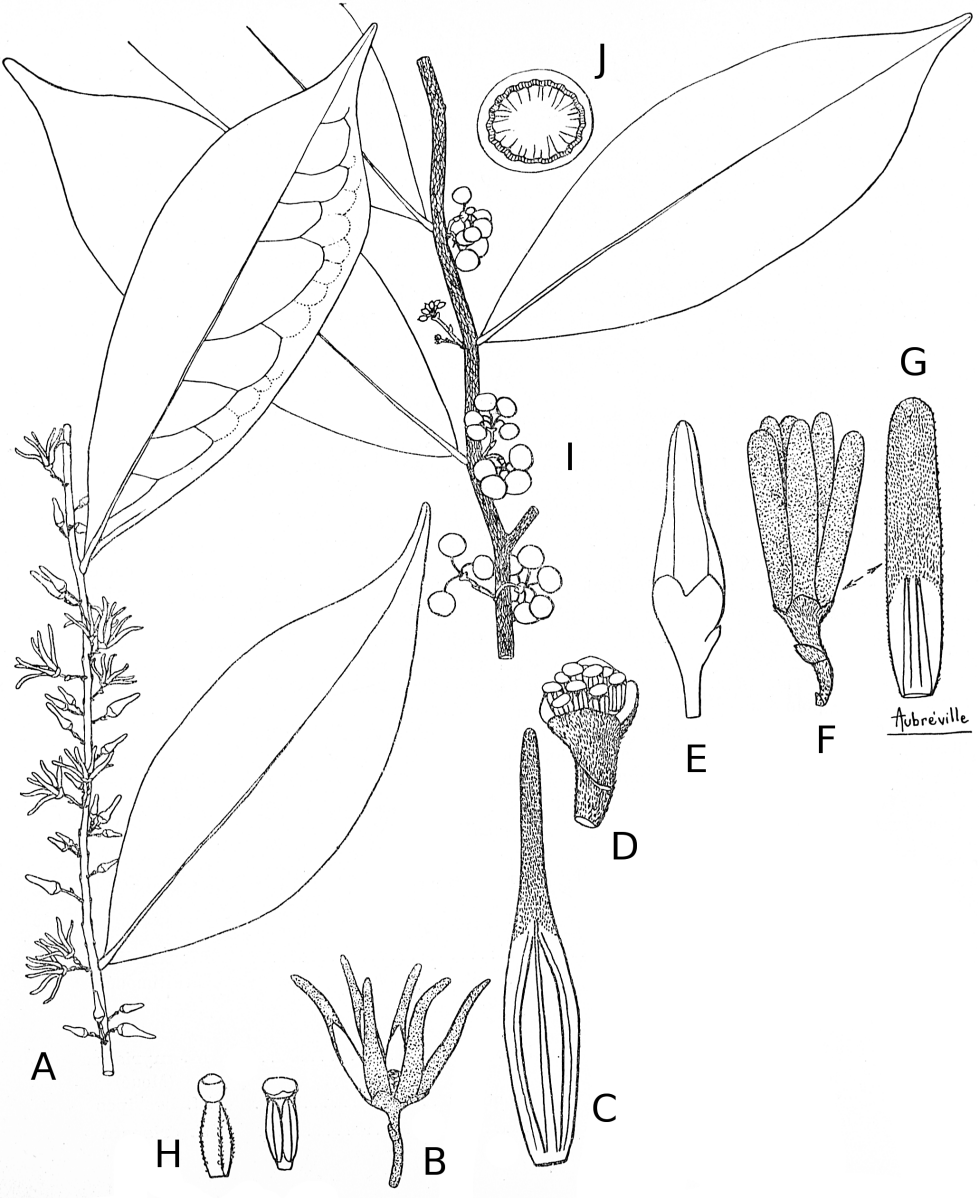
*Greenwayodendronoliveri*. **A** Leaves and inflorescence **B** Open flower **C** Inner petal **D** Androecium, male flower **E** Floral buds **F–G** Petals of a flower bud **H** Carpels and stamens **I** Leaves and fruits **J** Fruit section. Aubréville A (1936) *La flore forestière de la Côte d’Ivoire*, Volume 2. Larose, Paris, 296 p.

Tree 5–10 m tall, d.b.h. 3–25 cm. Young branches sparsely pubescent to glabrous, trichomes 0.1–0.2 mm long; old branches glabrous; Leaves: petiole 2.0–5.3 mm long, 1.0–2.3 mm in diameter, sparsely pubescent to glabrous, trichomes 0.1–0.2 mm long, indumenta brown; lamina 4.2–15.4 cm long, 2.0–6.1 cm wide, length: width ratio 1.5–3.4; elliptic to narrowly elliptic, base rounded or cuneate, apex acuminate, apiculate, aristate or caudate, acumen 1–33 mm long, upper and lower side sparsely pubescent; midrib pubescent to sparsely pubescent to glabrous on the upper side, pubescent to glabrous on the lower side, trichomes 0.1–0.4 mm long, brown; secondary veins 5–6 pairs, upper side glabrous, lower side pubescent to sparsely pubescent, trichomes ca. 0.1 mm long; tertiary veins irregular. Inflorescence axillary, a 1–4 flowered rhipidium. Floral buds ellipsoid, 5.8–7.5 mm long, 3.1–5.0 mm in diameter, densely pubescent; young pedicel densely pubescent. Flowering pedicel 3.8–6.2 mm long, 0.5–1.3 mm in diameter, densely to sparsely pubescent, trichomes 0.2–0.3 mm long, lower bract (on?) lower haft of pedicel, minute densely pubescent; upper bract apical, just below the calyx, 1.2–1.5 mm in diameter, densely pubescent, trichomes 0.2–0.3 mm long. Sepals 1.3–2.6 mm long, 1.6–3.2 mm wide, length:width ratio 0.5–0.9 broadly ovate, imbricate, fused at the base, apex acuminate, base truncate, outside pubescent, inside sparsely pubescent, trichomes 0.1–0.3 mm long. Inner and outer petals 8–18 mm long, 1.5–2.2 mm wide, length:width ratio 0.5–0.9, narrowly ovate, twisted or not, apex elliptic acuminate, base rounded; green maturing pale yellow, outside tomentose, trichomes 0.1–0.3 mm long, inside sparsely pubescent to glabrous; glabrous part to 4.5–8.0 mm long. Male flowers: stamens 10–25, in several whorls, 1.4–2.2 mm long, 0.4–0.9 mm wide; connectives of the flattened stamen; hermaphrodite flowers: stamens 5–10 in a single whorl, appressed, 1.4–1.8 mm long and 0.3–0.7 mm wide, connective of the stamens crushed-flattened in shape; carpels 10–15, 1.3–2.1 mm long, 0.6–0.9 mm in diameter, length:width ratio 1.2–2.0 narrowly oblong, densely pubescent; ovules 1–2, oblong; stigmata ovoid, densely pubescent, trichomes ca. 0.3 mm long. Fruiting pedicel 6–13 mm long, 1–2 mm in diameter, sparsely pubescent, trichomes ca. 0.4 mm long; stipes 7.5–8.3 mm long, 1.0–2.1 mm in diameter; monocarps 4–8, 3.0–8.2 mm in diameter, broadly ellipsoid to globose, sparsely pubescent, green turning wine red at maturity; seeds 1 per monocarp, 2.8–6.9 mm in diameter, ellipsoid to globose, flattened when more than one seed per monocarp, surface covered by a white tegument.

##### Distribution.

**Figure 8.**
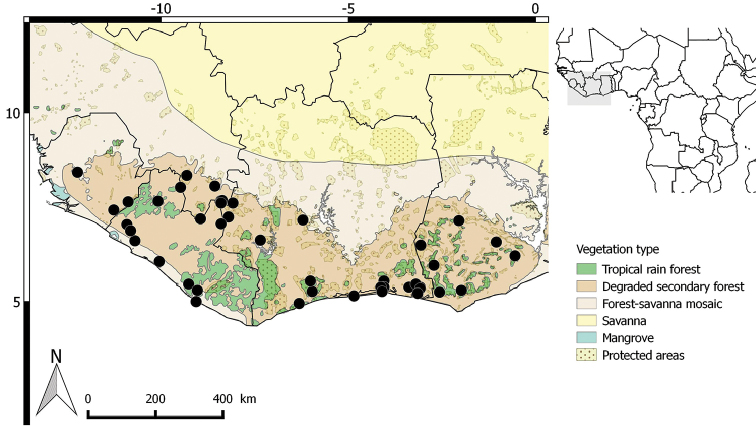
Distribution of *Greenwayodendronoliveri*.

Occurs in Ivory Coast, Guinea Conakry, Ghana, Liberia and Sierra Leone; 55–864 m (Fig. 8).

##### Habitat and ecology.

In moist and semi-deciduous forests.

##### Phenology.

In Ivory Coast and Ghana, flowering from February to April. Fruits are immature from May to September and fruits are mature from October to December.

##### Vernacular names.

**Ivory Coast**: Mpahouéfon (Abé), Baouéfou, **Sierra Leone.** Gatema (Mendé).

##### Uses.

Unknown.

##### Preliminary conservation status of IUCN.

Least Concern [LC]. The extent of occurrence (EOO) of *Greenwayodendronoliveri* is estimated to be over 260,482,084 km^2^, whereas its area of occupancy (AOO) is estimated to be 136 km^2^ (which falls within the limits for Endangered status under criterion B2). The species, recorded from five countries (Ivory Coast, Guinea Conakry, Ghana, Liberia and Sierra Leone), is now known from at least 34 specimens representing 26 subpopulations. These 26 subpopulations represent 22 different locations (sensu IUCN 2012), many more than 10 locations, which are the upper limit for Vulnerable status under subcriterion ‘a’. *Greenwayodendronoliveri* has been collected in 10 protected areas in the following countries (Ivory Coast, Guinea Conakry, Ghana, Liberia and Sierra Leone). The main threat to *G.oliveri* is its habitat destruction resulting from logging activities and intensive agriculture in West Africa. Notwithstanding these human activities, with varying levels of impact, the species appears not as threatened as it is abundant. The available information suggests that the number of subpopulations and mature individuals of *G.oliveri*, as well as its EOO and AOO, will not decrease noticeably in the next ten years.

##### Notes.

This species closely resembles *G.littorale*, see under that species for more details.

##### Selected specimens examined.

**Ghana. Eastern Region**: Atewa Range Forest Reserve, along footpath going uphill from Apapam to the South, 6°4.8'N, 0°21.6'W, 20 Oct 1994, *Jongkind, C.C.H. 1795* (P); Atewa range Forest Reserve: ca. 2.0 km S of the town of Asiakwa, 6°8.142'N, 0°19.86'W, 15 Nov 1995, *Schmidt, H.H. 1706* (WAG). **Western Region**: Pra Suhien Forest Reserve, 5°15'N, 2°36'W, 10 Nov 1971, *Deaw, J. 371* (WAG); Dunkwa Dist, Denyau Forest Reserve, 7°9'N, 2°30'W, 19 Feb 1963, *Enti, A.A. 7812* (WAG); Ankasa R.R, 5°35.30'N, 2°26.097'W, 22 Feb 2013, *Hawthorn, H.C. 339* (BRLU).

**Guinea. Nzérékoré**: Nimba Montains, SMFG iron ore mine concession, Gouan River valley, 7°41.32'N, 8°23.17'W, 14 Oct 2011, *Bilivogui, D. 116* (P); Nimba Mountains, Gouan Valley, 7°41.40'N, 8°22.90'W, 21 Aug 2008, *Jongkind, C.C.H. 8378* (WAG).

**Ivory Coast. Bas-Sassadra**: Tai – Grabe, forest at W Mono, 5°31'N, 7°19'W, 23 Mar 1969, *Bamps, P. 2225* (BR); 35 km SW of Guéyo, 5°33'N, 6°10'W, 27 Mar 1962, *Leeuwenberg, A.J.M. 3742* (BR, P). **Lagunes**: Abidjan, Banco Forest Reserve, south of Arboretum, 5°22'N, 4°30'W, 20 Jul 1973, *Koning, J. de 195*3 (WAG); Baouéfou. Banco, 5°23.40'N, 4°3.07'W, 15 Jan 1931, *Martineau 318* (P); ca. 5 km SE of O.R.S.T.O.M. Ile Boulay beyond lagune Ebrié, 5°16'N, 4°60'W, 22 Jul 1963, sandy soil, *Wilde, W.J.J.O. de 51*0 (BR, P); Pinhou, Plantation planche, 6°38.16'N, 7°21.24'W, Mar 1970, *Bamps 2585* (MO, P). **Région Sud Comoé**: Ashanti, 6°35'N, 1°30'W, Jan 1951, *Andoh, J.E. 5458* (BR); Forêt d’Anguédédou, 5°23'N, 4°80'W, 5 Sep 1969, *Thijssen, M.T. 317* (P).

**Liberia. Bomi**: Gola Forest NE of Bomi Hills, forest just outside the National Forest, 6°53'N, 10°49'W, 29 Apr 1966, *Bos, J.J. 1953* (BR, P, WAG); Place, 32 km W of Bomi Hills, road to Mano, 6°53.21'N, 10°49.39'W, 12 Nov 1969, *Jansen, J.W.A. 1509* (U). **Grand Bassa**: 32 km N of Buchanan, near waterfalls in the Zoh River (Bassa dial.), 6°40'N, 10°30'W, 20 Nov 1970, *Jansen, J.W.A. 1890* (WAG). **Gbarpolu**: Kpelle National Forest, 93 km E of Bopolu, 7°40'N, 10°50'W, 18 Jan 1978, *Gier, A. de. 205* (WAG); South-west of Togba Ville, 5°28.6'N, 9°16.3'W, 2 Dec 2012, *Jongkind, C.C.H. 10018* (WAG). **Grand Gedeh**: Mim Timber Co (Fijnhout), 5°18'N, 9°2'W, 14 May 1970, *Koning, J. de 453* (WAG); Eastern Province, Putu District. Near the village of Kanweake, ca. 70 km S of Chiehn (Zwedru village), 7°37'N, 8°50'W, 1962, *Wilde, J.J.F.E. de 10083* (P). **Nimba**: Nimba Mountains, 7°12'N, 8°57'W, 10 Apr 1962, V*oorhoeve, A.G.O.1073* (BR, WAG).

**Sierra Leone. Eastern Province**: Gola National Park, central block. East of Malimbe Camp, 7°39'N, 10°53'W, 23 Oct 2013, *Burgt, X.M. van der. 1612* (P); **Southern Province**: Yoni bani, 8°26.37'N, 12°14.22'W, 11 Nov 1914, *Thomas, N.W. 5059* (BR).

#### Greenwayodendron suaveolens

Taxon classificationPlantaeMagnolialesAnnonaceae

(Engl. & Diels) Verdc. Adansonia, n.s. 9: 90. 1969;

Figs 9 , 10

Polyalthia suaveolens Engl. & Diels, Monogr. Afr. Pfl. 6: 42. (**1901**). **Type. GABON. Estuaire**: Munda Sibange Farm, 20 Feb 1881, *H. Soyaux 218* (holotype material presumably destroyed at B†; lectotype, here designated: P![P00363356]; isolectotype K![K000580898]).
Polyalthia mortehanii De Wild., Bulletin Jardin Botanique. État Bruxelles, 4: 384. (1914). **Type. DEMOCRATIC REPUBLIC OF CONGO**. **Kasaï-Oriental**: Lekimi, Dec 1913, *S. De Giorgi 1576* (lectotype, here designated: BR![BR8804408]).
Polyalthia aubrevillei Ghesquière ex Aubréville, Fl. For. Côte d’Ivoire, i. 114 (1936). **Type. CAMEROON. South Region**: Bipindé, Urwaldgebiet, 1913, *G. Zenker*, 1306 (lectotype, here designated: P![P01985238]; isolectotypes: L web [L.1761577], MO web, P web [P01985239], WAG web [WAG.1379971].
Maba gossweileri Greves., J. Bot. 67 (Suppl. 2): 76. (1929). **Type. ANGOLA. Cabinda**: Buco Zau - Maiombe, 8 Jan 1917, *J. Gossweiler* 6923 (holotype BM web [BM000547162]; isotype COI! [COI00004858].
Xylopia otunga Exell., J. Bot. 69: 99 (1931). **Type. CAMEROON. Central**: Bitye Yaoundé, 1919, *G.L. Bates 1226* (holotype: BM web [000513697], isotype LISC web [LISC000385]).

##### Description.

Tree 8–45 m tall, d.b.h. 10–125 cm. Young branches at first sparsely pubescent, later glabrous, trichomes 0.2–0.8 mm long, erect; old branches glabrous. Leaves: petiole 2–8 mm long, 1.0–2.5 mm in diameter, pubescent to glabrous, trichomes 0.3–0.8 mm long, indumenta brown; lamina 5.1–15.6 cm long, 2.0–6.7 cm wide; length:width ratio 1.5–4.0; elliptic to narrowly elliptic, base rounded or cuneate, apex acuminate, apiculate, aristate or caudate, acumen 6–14 mm long, upper side glabrous, lower side densely to sparsely pubescent; midrib upper side basely sparsely pubescent, lower side densely to sparsely pubescent, trichomes 0.4–0.8 mm long; secondary veins 5–12 pairs, upper side glabrous, lower side pubescent to sparsely pubescent, trichomes 0.3–0.8 mm long; tertiary veins irregularly prominent, slightly raised or indistinct above. Inflorescence 1–4 flowered per rhipidium. Floral buds ellipsoid, 4–8 mm long, 3.0–4.6 mm in diameter, densely pubescent. Flowers, flowering pedicel 3.0–6.3 mm long, 0.8–2.1 mm wide, trichomes ca. 0.5 mm long, bract 1.4–3.1 mm in diameter, pubescent, trichomes 0.4–0.5 mm long. Sepals 1.8–3.8 mm long, 2.1–3.9 mm wide, length:width ratio 0.5–0.9 broadly ovate, apex acuminate, base truncate, outside pubescent, inside sparsely pubescent towards the centre, trichomes 0.1–0.5 mm long. Inner and outer petals 8–18 mm long, 1.3–2.6 mm wide, length: width ratio 0.7–0.9, narrowly ovate, to narrowly elliptic, apex acuminate, base rounded, outside pubescent, trichomes 0.2–0.5 mm long tomentose, erect, inside sparsely pubescent to glabrous, glabrous part to 2.0–4.1 mm long green maturing pale yellow. Male flowers: stamens 16–25, in several whorls, 2–4 mm long, 0.4–0.8 mm wide, tightly appressed; connectives tongue-shaped; hermaphrodite flowers: stamens 5–10 in a single whorl, appressed, 0.9–2.2 mm long and 0.3–0.8 mm wide, connective tongue-shaped; carpels 12–20, 0.7–1.6 mm long, 0.5–0.8 mm wide, length:width ratio 2.3–2.7 oblong, densely pubescent; ovules 1–2, oblong; stigmata ovoid, densely pubescent, trichomes ca. 0.5 mm long. Fruiting pedicel 5.5–12.0 mm long, 1.5–2.5 mm wide, sparsely pubescent, trichomes ca. 0.5 mm long; stipes 4.5–10.0 mm long and 1.0–2.5 mm wide, sparsely pubescent; monocarps 2–8, 7.2–16.4 mm in diameter, broadly ellipsoid to globose, sparsely pubescent to glabrous, green turning wine red at maturity; seeds 1–4 per monocarp, 3.0–11.2 mm in diameter, ellipsoid to globose, flattened when more than one seed per monocarp, surface covered by a white tegument.

**Figure 9.**
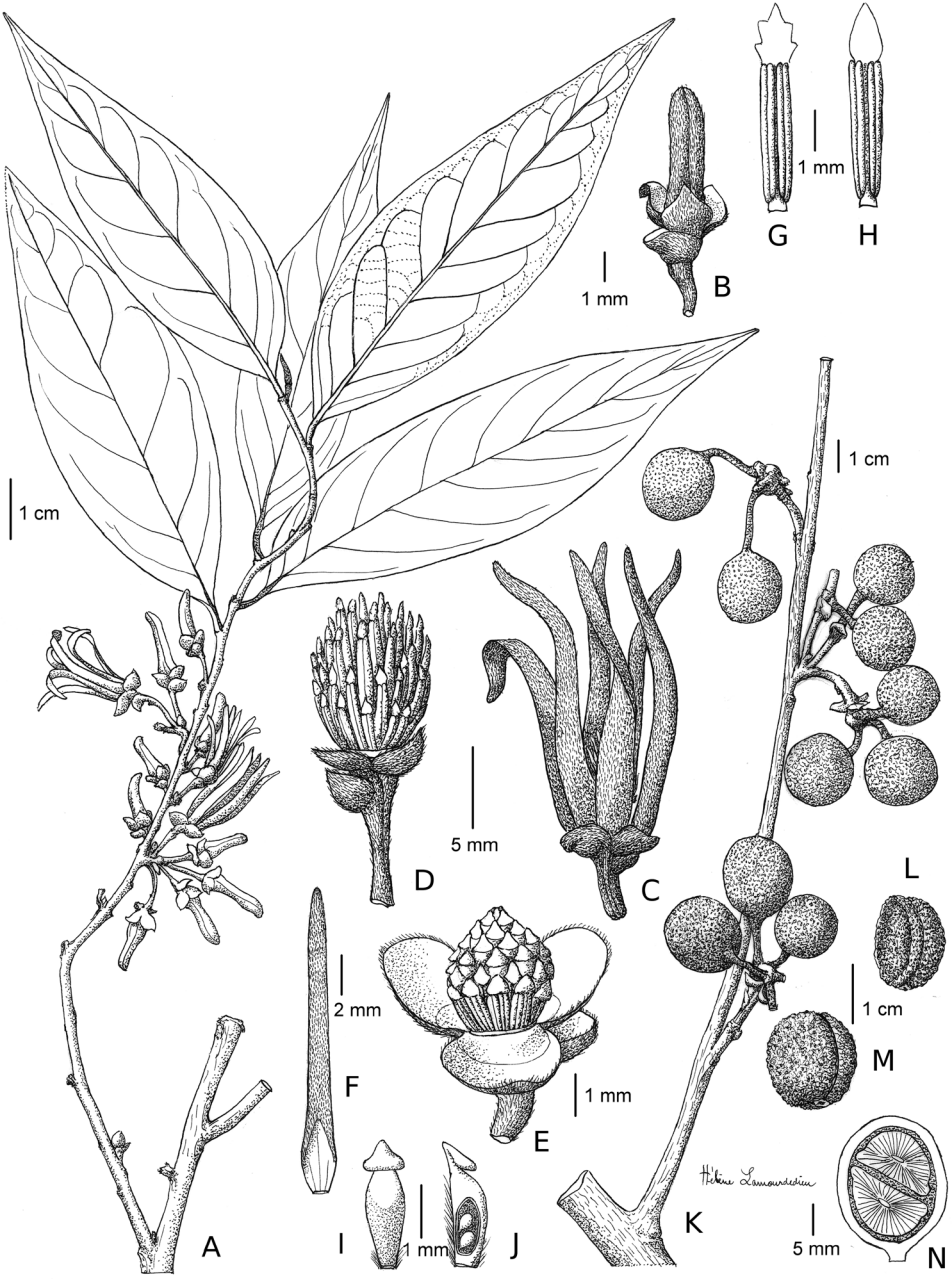
Greenwayodendronsuaveolens. **A** Flowering branch **B** Flower bud **C** Flower at anthesis **D** Detail of male receptacle, petals removed **E** detail of hermaphrodite receptacle, petals removed **F** Inside view of outer petal **G** Stamen **H** Stamen **I** Carpel **J** Longitudinal section of carpel **K** Fruiting branch **L** Lateral view of seed **M** Seed **N** Longitudinal section of a single monocarp showing two seeds and their ruminations. Drawings Helène Lamourdedieu, © Publications Scientifiques du Muséum national d’Histoire naturelle, Paris; modified from Le Thomas (1969: 205, pl. 37). **A–D, F–H***Le Testu 9408***E, I, J***Gilbert 936***K–N***Letouzey 5322*.

**Figure 10.**
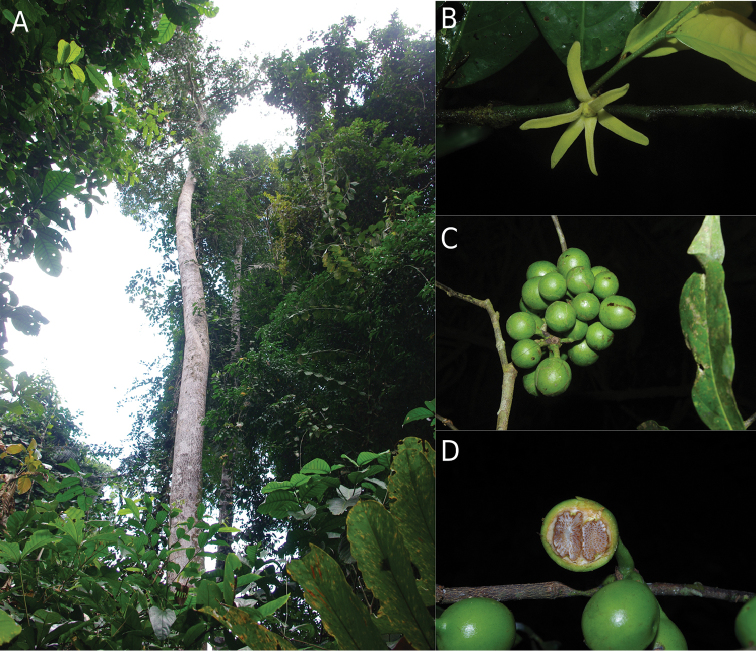
Greenwayondendronsuaveolens. **A** Habit **B** Flower **C** Fruits and monocarps **D** Latitudinal section of one monocarp showing ruminate endosperm of seeds. Photos: TLP Couvreur.

##### Distribution.

**Figure 11.**
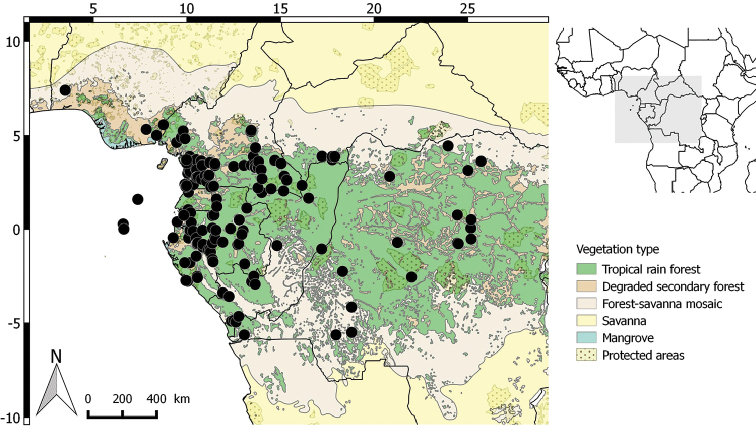
Distribution of Greenwayodendronsuaveolens.

Widespread across Central Africa, in Nigeria, Cameroon, Republic of Congo, Gabon, Equatorial Guinea (Rio Muni), Central African Republic, Uganda, São Tomé-and-Prìncipe, Democratic Republic of the Congo, Angola (Cabinda) (Fig. 11).

##### Habitat and ecology.

Moist evergreen and semi-deciduous lowland and mid-altitude 30–1600 m in forests.

##### Phenology.

In Gabon and southwest Cameroon, flowering from January to April. Fruits are mature from November to December.

##### Vernacular names.

**Cameroon: Moabé noir** (Nzime), Otunga (Fang), Otungui (Ewondo) Ntoulen (bassa), Botounga (Baka). **Gabon**: Mutunga (Aduma, Awandji and Nzebi), Otunga (Fang, Kota and Obamba). **Democratic Republic of Congo**: Yako-Ledale, Babua Embaye, Bombai Bo Ilo (Turumbu), Mwamba (Kiumba), Moamba Ndombe, Bombaye, Djako-Ledale. **Central African Republic**: Modienge (Lissango), Motunga. **Nigeria**: Nchua (Bokyi), EDO Ewáé (Edo), Eleku (Isekiri) okeren (Kennedy) Atorewa (Urhobo), agudugbu (Yoruba).

##### Uses.

The wood of Greenwayodendronsuaveolens is used for carpentry and construction of habitats, but also for the manufacture of hunting and fishing spears (Le Thomas 1969). Bark decoctions are used as a laxative to facilitate childbirth and as a stimulant for women’s fertility in the Republic of Congo (Boutique 1951). In Cameroon and Gabon, bark ash is rubbed in scarifications, on the forehead to treat psychosis and bark paste is applied externally to treat rheumatism, headache, epilepsy, toothache and malaria (Le Thomas 1965, Raponda-Walker and Silians 1961). In the Democratic Republic of Congo, decoctions of the bark are used to calm colic. In Nigeria, the leaf has been recorded as being taken internally for menorrhagia.

##### Preliminary IUCN conservation status.

Least Concern [LC]. The extent of occurrence (EOO) of Greenwayodendronsuaveolens is estimated to be over 2,316,419 km^2^, whereas its area of occupancy (AOO) is estimated to be 476 km^2^ (which falls within the limits for Vulnerable status under criterion B2). The species, recorded from 10 countries (Nigeria, Cameroon, Republic of Congo, Gabon, Equatorial Guinea (Rio Muni), Central African Republic, Uganda, Sao Tome and Principe, Democratic Republic of Congo and Angola (Cabinda)), is now known from at least 126 specimens representing 88 subpopulations. These 88 subpopulations represent 93 different locations (sensu IUCN 2012), many more than 10 locations, which are the upper limit for Vulnerable status under subcriterion ‘a’. Greenwayodendronsuaveolens has been collected in 13 protected areas in the following countries: Nigeria, Cameroon, Republic of Congo, Gabon, Equatorial Guinea (Rio Muni), Central African Republic, Uganda, São Tomé-and-Prìncipe, Democratic Republic of the Congo, Angola (Cabinda). The main threat to G.suaveolens is its habitat destruction resulting from urbanisation in Central Africa as well as intensive agriculture and mining in the Democratic Republic of Congo. Notwithstanding these human activities, with varying levels of impact, the species appears not as threatened as it is abundant. The available information suggests that the number of subpopulations and mature individuals of G.suaveolens, as well as its EOO and AOO, will not decrease noticeably in the next ten years.

##### Notes.

In Gabon, G.suaveolens and G.gabonicum occupy the same habitat (mature *tierra firme* forest) and can occur in sympatry. However, G.gabonicum has a clearly tomentose pubescence of the lower face of the lamina which is sparsely pubescent in G.suaveolens.

In the original description of *P.? acumianata*, Oliver cited two syntypes (*Mann 841* and *Thomson 109*). However, both represent different species (Verdcourt 1969). The former is the type species of G.oliveri and the latter a specimen of G.suaveolens.

We lectotypified the type specimen of G.suaveolens, as the specimen *H. Soyaux* 218 from Berlin is thought to have been destroyed. In describing Polyalthiamortehani, De Wildeman (De Wildeman, 1914) did not designate a type specimen. The observation of the two specimens present in BR (*Mortehan 362*; *De Giorgi 1576*), led us to select the sample *De Giorgio* 1576 as the lectotype because it contains the best flowers’ information.

In their description of Polyalthiaoliveri, Engler and Diels (1901) identified the Zenker G.A. specimens from Cameroon as P.oliveri. In the Flore Forestière de la Côte d’Ivoire (1936), Aubréville indicates that these Zenker specimens are not P.oliveri and introduces the name P.aubrevillei (now a synonym of G.suaveolens). He refers to the illustration of Engler and Diels (1901) of P.oliveri as being Polyalthiaaubrevillei. He provided a key to separate both species (P.oliveri and P.aubrevillei) with “Etamines à connectif très allongé aigu” [Stamens with very long and acute connective] for *P. aubrévillei*. Following the code (Art. 38.13, McNeill et al. 2012), this description is valid because it contains a diagnose (the key) and was published prior to 1953 validating the direct reference of a previous description for the specimens belonging to this new name. Based on the specimens cited by Engler and Diels (1901) from Cameroon, we selected *Zenker 1306* as the lectotype because it contains numerous opened flowers.

Finally, it must be noted that analyses of morphology and genetic diversity (Lissambou et al. in press) identified a group of specimens collected in São Tomé and Prìncipe as possibly distinct. However, to date, the status of this group of specimens is inconclusive, partly related to the lack of fertile material.

##### Selected specimens examined.

**Angola. Luali – Buco Zan**: Ponga Mungo-Subluali, 4°39.10'S, 12°46.10'E, 14 Feb 1916, *J. Gossweiler.* 6275 (COI); Maiombe – Subluali, 4°39.11'S, 12°46.15'E, 31 Sep 1916, *J. Gossweiler* 6229 (COI); Ponga Mungo – Subluali Maiombe, 14 Jan 1916, *J. Gossweiler* 6145 (COI).

**Cameroon. Central**: 2 km NW of Ossoéssam (Village among Rhaphia) about 40 km SSW Of Mbalmayo, 3°25'N, 11°30'E, 1 Apr 1965, *Leeuwenberg, A.J.M. 5755* (BR, P); 41 km from the LBC Eseka sawmill, 3°39'N, 10°46'E, 14 Feb 1953, *Mpom, B. 74* (P, YA); Forêt classée de Mbalmayo, 3°43'N, 9°59'E, 15 Nov 1970, *Mpom, B. 537* (P, YA); Riverine forest. Bank Nyong Rivers, near the new bridge, about 65 km SSW of Eséka, 3°39'N, 10°46'E, 17 Jul 1964, *Wilde, W.J.J.O. de. 2857* (BR; P). **East region**: Around the village of Mindourou I. UFA managed by Pallisco, 3°16.464'N, 13°23.01'E, 9 Nov 2012, *Droissart, V. 1420* (BRLU); 15 E of Dimako, 4°21'N, 13°40'E, 11 Dec 1965, *Leeuwenberg, A.J.M. 7322* (BR, P, WAG); Colline de l’ENE de Mbalam (140 km) of ESE Djoum, near Souanke-Congo, 2°13'N, 13°48'E, 20 Jan 1973, *Letouzey, R. 11866* (P, YA, WAG); UFA 10039B Palisco Mindourou, 3°26.0712'N, 13°26.081'E, 12 Mar 2016, *Lissambou, B.J. 2100* (BRLU). **Littoral**: 20 km from Kribi lumbering, 3°00'N, 10°03'E, 9 Jun 1970, *Bos, J.J. 4769* (BR, P, YA, WAG); EDEA/Village Pout-loloma, 3°53.041'N, 10°8.824'E, 15 Jan 2016, *Lissambou, B.J. 1725* (BRLU); EDEA/Village Pout-loloma, 16 Jan 2016, *Lissambou, B.J. 1726* (BRLU). **South Region**: La lobé, 0°32'N, 12°46'E, 2 Dec 1927, *Hedin 1943* (BR); Ebolowa-Ambam, 16 km on the road from Ebolowa to Minkok, 2°58'N, 11°17'E, 12 Sep 1975, *Wilde, J.J.F.E de. 8465* (BR, YA, P); 13 km along the road from Kribi to Ebolowa. Raphia swamp with small creek in the middle, 2°52'N, 10°00'E, 26 Nov 1975, *Wilde, J.J.F.E. de 8674* (P, YA, WAG); 10 km environ à l’ESE de Campo Kribi, 2°15'N, 9°60'E, 26 Mar 1968, *Letouzey, R. 9198* (YA, P); Parc Campo MA’AN. Mature primary forest, 2°17.424'N, 9°56.893'E, 18 Jan 2016, *Lissambou, B.J. 1795* (BRLU); Nkomekak, 2°46.990'N, 10°31.858'E, 5 Feb 2016, *Lissambou, B.J. 1900* (BRLU); Bipendi. Urwaldgebiet, 3°40'N, 10°24'E, 1900, *Zenker, G.A. 2166* (YA, P, WAG); Bipendi. Urwaldgebiet, 3°40'N, 10°34'E, 1912, *Zenker, G.A. 4435* (P, WAG). **South West region**: Near Ngombombeng village, north of Nyasoso, 4°54'N, 9°42'E, 31 Apr 1986, *Etuge, M. 30* (P, U, YA); Mile 12 Mamfé road between Kumba and Baduma, 4°45'N, 9°29'E, 4 Oct 1986, *Nemba, J. 291* (P, WAG).

**Central African Republic. Sangha-Mbaéré**: Réserve de Dzanga-Sangha. Lowland forest, 2°22'N, 16°10'E, 14 Oct 1988, *David, J. 1414* (BR); Grima (Commun de Ngotto), 3°54'N, 17°13'E, 11 Mar 1999, *Yangakola, J.M. 153* (BRLU); Ngotto, 3°54'N, 17°13'E, 26 Jul 1994, *Yalibanda, Y. 94/36* (BRLU). **Lobaye**: Boukoko, forest, 3°43'N, 17°46'E, 7 Oct 1948, *Tisserant, C. 1172* (BR, P); Boukoko, 3°43'N, 17°46'E, 25 Aug 1953, *Tisserant, R.P.* 2573 (P).

**Democratic Republic of the Congo. Kantanga**: Kiobo, 5°38'S, 13°07'E, 2 Apr 1944, *Donis, C. 84* (BR). **Kasaï-Oriental**: South o Booke (Terre, Kolo), 2°33'S, 22°00'E, May 1958, *Robin, R. 90* (BR); Dundusana, Lekimi, 2°53'N, 22°23'E, Dec 1913, *Mortehan 362* (BR). **Orientale**: Luki, vallée N’tosi, 5°38'S, 13°04'E, 14 Jul 1948, *Donis, C. 1916* (BR); Yangambi, 0°46'N, 24°27'E, May 1937, *Gilbert 96* (BR, P); Yangambi, Réserve flore Isalowe, 0°44'S, 24°27'E, 19 Aug 1938, *Louis, J. 10916* (MO, P).

**Equatorial Guinea. Rio Muni, Cento-Sur**: Mont Alén, 1°30'N, 10°04'E, 16 Jun 1998, *Ngomo, D. 342* (BRLU, MO); SO National Monte Alen Park, 200 m S of transct Ecofac of Mosumo 1620 early layon, 1°35'N, 10°03'E, 10 Mar 2011, *Senterre, B. 784* (BRLU, MO).

**Gabon. Estuaire**: Brigade forestière d’Ekouk (nouvelles parcelles), 15 km north of Koulounga, 0°45'N, 9°50'E, 22 Sep 1983, *Floret, JJ. 1389* (LBV, WAG); S of Ekouk, 0°6'S, 10°20'E, 2 Nov 1983, *Louis, A.M. 308* (LBV, WAG); Forest exploitation Leroy, in road construction area, 0°57'S, 10°52'E, 19 Jan 1983, *Wilde, J.J.F.E. 73* (LBV, WAG, U); Crystal Mountains, 0°53'S, 10°12'E, 20 km NW of Asok, Closed high forest in hilly country, 20 Jan 1983, *Wilde, J.J.F.E. 101* (LBV, WAG, P). **Moyen-Ogooué**: Mabounié, 45 km southwest of Lambaréné, north bank of the Ngounié River, 0°28.854'S, 10°18.846'E, 13 Nov 2013, *Bidault, E. 1297* (BRLU, LBV, MO). **Ngounié**: *ibid. loc.*, 0°26.706'S, 10°19.458'E, 13 Oct 2012, *Bidault, E. 798* (BR, BRLU, LBV, MO); Parc National de Waka, 1°06'S, 11°09'E, 7 Oct 2007, *Boussiengui Nongo, J. 259* (LVB); Between Mouila to Yeno about 60 km from Mouila, 1°44'S, 11°24'E, 21 Sep 1986, *Breteler, F.J. 8086* (BR, LBV, WAG); Massif du Chaillu, north-east of Mouila, Leroy shipyard, 1°40'S, 11°15'E, 24 Apr 1989, *McPherson, G.D. 13907* (BR, P). **Nyanga**: Chantier CEB, ca. 45 km SW of Doussala, 2°35'S, 10°34'E, 22 Oct 1985, *Reitsma, J.M. 1737* (LBV); Kwassa. Fishing village next to Banio Lagune, 3°23'S, 11°55'E, 13 May 2001, *Walters, G.M. 656* (LBV). **Ogooué-Ivindo**: Makande surroundings, ca. 65 km SSW of Booué, 0°41'S, 11°55'E, 9 Feb 1999, *Breteler, F.J. 14970* (LBV); Station de Recherche de l’Institut de Recherche en Ecologie tropicale (IRET-Ipassa), 0°29.808'N, 12°46.961'E, 1 May 2015, *Lissambou, B.J. 1299* (BRLU, LBV, MO); Réserve de la Lopé, south of Ayem, site SOFORGA, 00°25'S, 11°30'E, 10 Mar 1989, *McPherson, G.D 13748* (LBV); 25 km NE of Booué, 0°00'S, 12°20'E, 19 May 1987, *Wilks 1544* (MO, P). **Ogooué-Lolo**: ca. 30 km E of Lastoursville, 0°40'S, 13°00'E, 20 Nov 1991, *Breteler, F.J. 10611* (LBV); Forêt des Abeilles; 9 km S of Confluence Gongue-Offoue, 0°48'S, 11°45'E, 28 Jun 1996, *Wilks, C.M. 2695* (WAG); Région de Lastoursville, 0°49'S, 12°43'E, 12 Mar 1931, *Le Testu M.G. 8698* (BR, P). **Ogooué-Maritime**: Mont Doudou, Campagne, 2°31'S, 10°33'E, 19 Sep 2000, *Bourobou, H.P. 323* (LBV); Toucan, edge road, 1°47'S, 9°53'E, 8 Jun 2002, *Bourobou H.P. 704* (WAG); Lac Azingo. Grands lacs Moyen, 0°28.638'S, 10°1.992'E, 15 Oct 2014, *Lissambou, B.J. 0011* (BR, BRLU, LBV, MO, P, WAG). **Woleu-Ntem**: ca. 25 km WSW of Mintsic, inventory Oveng, 0°44'N, 11°22'E, 9 Feb 1987, *Reitsma, J.M. 2957* (LBV, WAG); ca 25 km WSW of Mintsic, Chantier Oveng, 0°44'N, 11°22'E, 9 Nov 1986, *Reitsma, J.M. 2580* (LBV); Inselberg Milobo, 0°56'N, 10°30.9'E, 8 Jul 2001, Ngok Banak, L. 45 (LBV); Near Essong, ± 5 km NW of Mitzic, along exploitation road, 0°46'N, 11°27'E, 9 Nov 1991, *Louis, A.M. 550* (LBV, P, WAG).

**Nigeria. South Eastern State**: Boshi extension Forest Reserve, 13°20'N, 9°20'W, 25 May 1971, *Van Meer, P.P.C. 1795* (WAG); Forest Reserve of Enyong, 5°20'N, 7°50'W, 8 Apr 1971, *Van Meer, P.P.C. 1228* (WAG). **Western State**: Abeokuta, 3°30'N, 7°25'W, *Wit, P. 2111* (K).

**São Tomé and Príncipe. Prìncipe**: Alentours de Zona ecológica. Pico Papagaio (Zona ecológica), 1°36.75'N, 7°391'E, 26 Mar 1998, *Oliveira F. de. 588* (BRLU, MO). **São Tomé. Mé-Zochi**: Bom Sucesso to Lagoa Amelia (site S.P, km 0,6), 0°7.02'N, 6°35'E, 4 Jul 1987, *Lejoly, J. 97/362* (BRLU, MO); Base Pico Maria Fernandes, 0°10'N, 6°38'E, 27 Feb 2003, *Ogonovszky, M. 293* (BRLU, MO); Lago Amelia, south of Bom Sucesso Botanical Garden, 0°16'N, 6°35'E, 11 Feb 2009, *Dauby, G.1574* (BRLU, MO).

**Republic of Congo. Kouilou**: Forest site at 20 km N of Loundji Moyombe, 0°52'S, 14°49'E, 23 Mar 1969, *Attims, Y. 115* (P); Douakani, 2°56.038'N, 13°8.975'E, *Kamit, T 1015* (K); Road to Boungolo site, Kakamoeka (Point-Noir). **Niari**: Lepoutou, 2°47.895'S, 13°27.52'E, 25 Jun 2011, *Mpandzou, A.L. 1104* (K).

#### Greenwayodendron usambaricum

Taxon classificationPlantaeMagnolialesAnnonaceae

(Verdc.) Lissambou, Hardy & Couvreur comb. nov.

urn:lsid:ipni.org:names:77192859-1

Greenwayodendron suaveolens subsp. usambaricum Verdc., Adansonia, sér. 2, 91. (1969).

##### Type.

**TANZANIA.** Tanga: Kwamkoro to Potwe. E. Usambaras, Dec 1936, *P.J. Greenway 4810* (lectotype, designated by Verdcourt 1969 p. 69, K![K000580892]; isotypes K![K000580893], EA).

Tree 12–30 m tall, d.b.h not observed. Young branches at first sparsely pubescent, later glabrous, trichomes 0.1–0.8 mm long, erect; old branches glabrous. Leaves: petiole 4.5–6.0 mm long, 1.5–2.1 mm in diameter, densely to sparsely pubescent, trichomes 0.1–0.8 mm long, indumenta brown; lamina 11.2–16.5 cm long, 4.1–6.4 cm wide; length:width ratio 2.3–3.0; elliptic to narrowly elliptic, base rounded or cuneate, apex acuminate, apiculate, acumen 10–25 mm long, upper side glabrous, lower side densely pubescent to sparsely pubescent; midrib upper side basely sparsely pubescent, lower side densely to sparsely pubescent, trichomes 0.3–0.7 mm long; secondary veins 14–18 pairs, upper side glabrous, lower side pubescent to sparsely pubescent, trichomes 0.3–0.8 mm long; tertiary veins irregularly prominent, slightly raised or indistinct above. Floral buds ellipsoid, 6–9 mm long, 2.0–3.2 mm in diameter, densely pubescent. Flowers not observed. Fruiting pedicel 12–13 mm long, 2.0–2.5 mm wide, sparsely pubescent, trichomes 0.4–0.5 mm long; stipes 5.5–6.5 mm long and 1.0–1.2 mm wide, sparsely pubescent; monocarps 2–4, 11.6–12.2 mm in diameter, broadly ellipsoid to globose, sparsely pubescent to glabrous, green turning wine red at maturity; seeds 1–2 per monocarp, 11.0–11.2 mm in diameter, ellipsoid to globose, flattened on one side when more than one seed per monocarp, surface covered by a white tegument.

##### Distribution.

**Figure 12.**
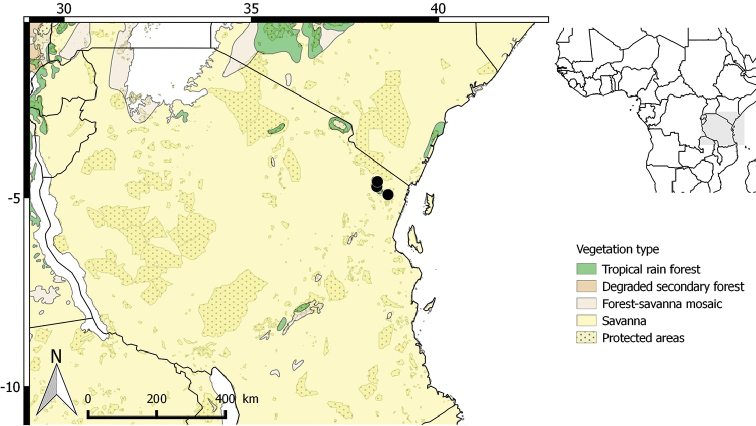
Distribution of *Greenwayodendronusambaricum*.

Only known from northern Tanzania, in the East Usambara Mountains, 320–1124 m (Fig. 12).

##### Habitat and ecology.

Mature forest in mid-altitude mountain.

##### Phenology.

Flowering and fruiting are not well known. Nevertheless, floral buds samples were collected in November and December. Fruit specimens were collected from August to November.

##### Vernacular names.

Unknown.

##### Uses.

Unknown.

##### Preliminary IUCN conservation status.

Vulnerable [VU]. The extent of occurrence (EOO) of *Greenwayodendronusambaricum* is estimated to be over 2 km^2^ (within the 20,000 km^2^ upper limit for Endangered status under criterion B1) whereas its minimal area of occupancy (AOO) is estimated to be 36 km^2^ which falls within the limits for Endangered status under criterion B2. *Greenwayodendronusambaricum* is endemic to the Usambara Mountains in Tanzania where it is dominated under canopy. It has been collected in one protected area (Mount Usambara). This species is known from 9 specimens representing 7 subpopulations. These 7 subpopulations represent a total of 7 “locations” (*sensu*IUCN 2012), exceeding the upper limit for Endangered status, but falling within the limit for Vulnerable status. The main *G.usambaricum* threat is habitat destruction resulting from logging and intensive agriculture in Tanzania. We project that the ongoing loss of its habitat will induce a strong continuous decline in the number of subpopulations and mature individuals in the next ten years as well as an important decline of its EOO and AOO. *Greenwayodendronusambaricum* is therefore assigned a preliminary status of VU B1ab(i,ii,iii,iv,v) + B2ab(i,ii,iii,iv,v).

##### Notes.

Initially, individuals from the Usambara Mountains in Tanzania were considered part of *G.oliveri*. Indeed, Engler and Diels (1901) cited the specimen *Scheffler 74* (Usambaras) under *Polylathiaoliveri*. Verdcourt (1969) then described the Usambara specimens as a subspecies of *G.suaveolens*, recognising little morphological differentiation with *G.suaveolens*. Here, we consider these individuals as representing a separate species from *G.suaveolens*. Indeed, besides its disjunct and isolated distribution, *G.usambaricum* can be distinguished from *G.suaveolens* by the higher number of secondary veins (≥ 14 versus ≤ 12 for *G.suaveolens*). Nevertheless, more flowering material is needed to better understand the morphological differences between these two species. These observations are corroborated to a certain extent by population genetic data such as microsatellites (Lissambou et al. in prep.) and especially nuclear phylogenetic analyses of the genus (Couvreur et al. in prep.). In the latter case, *G.usambaricum* was reconstructed as sister with maximum support to a clade containing *G.gabonicum, G.glabrum, G. litorale* and *G.suaveolens*.

##### Specimens examined.

**Tanzania**. **Tanga**: Bomole, Amani, hill trail ca. halfway to summit in area of natural forest, 5°02'S, 39°10'E, 4 Jun 1996, *Johnson, D.M. 1943*. Bomole, Amani Nature Reserve. East Usambara Mountains. Mature forest, 4°55'S, 38°36'E, 10 Oct 2007, *Marshall, A.R. 1260* (K); Bomole, Amani Nature Reserve. East Usambara Mountains, 4°55'S, 38°36'E, 8 Aug 2007, *Marshall, A.R. 1094* (K). Usambara, Afrika, Landsaft, 4°42'S, 38°21'E, 29 Aug 1916, *Peter, A.* 17595 (K, WAG); Usambara, Afrika, Landsaft, 4°42'S, 38°21'E, 29 Aug 1916, *Peter, A.* 18130 (K, WAG); Tanga. Kwamkoro, 4°42'S, 38°21'E, 11 Jun 1986, *Ruffo, A.* 2195 (K); Tanga. Kwamkoro F.R, 4°42'S, 38°21'E, 4 Aug 1961, *Sensé, SR. 3238* (BR).

### Excluded names

*Polyalthia? acuminata* Oliver (Oliver 1868): This name is not validly published as the name was already taken (*Polylathiaacuminata* Thwaites, a species from South East Asia)

Polyalthiaoliveri(Engl.)var.gabonica Pellegrin, Bull. Sco. Bot. Fr., Mém. 31: 67. 1949: *nomen nudum*, no Latin diagnosis.

### Index to numbered seen specimens (or Exsiccatae)

Akpabla, G.K. 849 (*oliveri*)

Aubréville, A. 168, 1107 (*oliveri*)

Andoh, J.E. 5458 (*oliveri*)

Attims, Y. 115 (*suaveolens*)

Baldwin J.T. 10710, 10802 (*oliveri*)

Bamps, C. 2240, 2150 (*oliveri*)

Bamps, P.R.J. 2225, 2267, 2394, 2585 (*oliveri*)

Bastin 81, 89, 189 (*suaveolens*)

Betti, J.L. 3, 95/67 (*suaveolens*)

Bidault, E. 800, 1297, 2116 (*gabonicum*); 576, 847 (*glabrum*); 798 (*suaveolens*); 300 (*oliveri*)

Bilivogui, D. 116 (*oliveri*)

Binuyo, A. 35623 (*suaveolens*)

Bos, J.J. 6267 (*glabrum*); 1953 (*oliveri*); 4769, 6100, 6267, 6289 (*suaveolens*)

Bourobou, H.P. 703 (*gabonicum*); 323, 704 (*suaveolens*)

Boussiengui Nongo, J. 259 (*suaveolens*)

Breteler, F.J. 14259, 15037 (*gabonicum*); 5649, 5912, 14481 (*littorale*); 5649, 5912, 7446, 13359 (*oliveri*); 649, 5743, 8086, 10611, 11090, 14970, 15492 (*suaveolens*)

Burgt, X.M. van der 1612 (*oliveri*)

Carette 21 (*suaveolens*)

Cheek, M.R. 27 66, 78, 343, 9088, 11224 (*suaveolens*)

Chevalier, A.J.B. 17821, 33069 (*oliveri*)

Couvreur, T.L.P. 540, 746, 888, 921, 1080, 1097 (*gabonicum*); 476, 560, 658, 703, 746, 749, 756, 857, 873, 966, 1002, 1082 (*suaveolens*)

Dauby, G.V. 603, 909, 865, 2331, 2809 (*gabonicum*); 2673 (*glabrum*); 313, 480, 871, 1145, 1574 (*suaveolens*)

David, J. 1414 (*suaveolens*)

De Giorgi, S. 1576 (*suaveolens*)

Deaw, J. 371 (*oliveri*)

Debroux 99 (*suaveolens*)

Dessein, S. 1948, 2102, 2228, 2277, 2309, 2466, 2507 (*suaveolens*)

Donis, C. 84, 1916, 2388, 2885, 2911, 2944 (*suaveolens*)

Droissart, V. 834, 1420, 2050, 2159 (*suaveolens*)

Enti, A.A. 1100, 7812 (*oliveri*)

Etuge, M. 30, 4452, 5010 (*suaveolens*)

Evrard, C.M. 40 (*suaveolens*)

Farron, C. 4896 (*suaveolens*)

Fleury, F. 33069 (*oliveri*)

Floret, J.J. 1531, 1788 (*glabrum*);1389, 1490 (*suaveolens*)

Fofana, F. 120 (*suaveolens*)

Foury, P 129 (*suaveolens*)

Geerling, C. 2342 (*oliveri*)

Gentry, A.H. 33185 (*suaveolens*)

Gerard, P. 2474 (*suaveolens*)

Gesnot, K. 8, 165 (*gabonicum*)

Gier, A. de 205 (*oliveri*)

Gilbert 36, 96, 1450, 8041, 8098, 8115, 8500 (*suaveolens*)

Gossweiler, J. 6145, 6229, 6275, 6505, 6923, 6982, 7186 (*suaveolens*)

Greenway, P.J. 4810 (*usambaricum*)

Haba, O.-.O. 1 (*oliveri*)

Hallé, N. 4053 (*suaveolens*)

Harries, D. 1482 (*suaveolens*)

Hawthorn, H.C. 142, 339 (*oliveri*)

Heuertz, M. 2676 (*suaveolens*)

Heutsz, J.B. van 2563 (*suaveolens*)

Hladik, A. 1855 (*suaveolens*)

Hoshino, J. 13, 14 (*glabrum*)

IRD Plot 91, 1283 (*gabonicum*); 25, 165, 406, 410, 430, 652, 714, 906, 970, 1011, 1018, 1125, 1206, 1217, 1393, 1453, 1507, 1624, 1796 (*suaveolens*)

Jansen, J.W.A. 1509, 1890 (*oliveri*)

Jongkind, C.C.H. 1795, 8378, 9486, 10018, 11561 (*oliveri*)

Kaji, M. 4 (*glabrum*); 20 (*suaveolens*)

Kami, T. 1015 (*suaveolens*)

Koning, J. 453, 4011, 1556, 1953 (*oliveri*)

Kruif, A.P.M. de 208 (*oliveri*); 997 (*suaveolens*)

Lachenaud, O.L.S. 1926, 1958 (*gabonicum*)

Le Testu, G.M.P.C. 7936 (*gabonicum*); 8698, 9408 (*suaveolens*)

Lebrun, J.-.P. 2407 (*suaveolens*)

Leeuwenberg, A.J.M. 5582, 12286, 3742 (*oliveri*); 5082, 5755, 7322, 8164 (*suaveolens*)

Lejoly, J. 97/362, 2700 (*suaveolens*)

Letouzey, R. 12869 (*glabrum*); 1106, 1190, 1214, 1304, 1313, 1339, 1877, 5322, 9198, 10360, 10456, 10656, 11866 (*suaveolens*)

Lissambou, B.J. 1, 2, 3, 4, 5, 7, 8, 11, 12, 300, 304, 332, 335, 353, 1134, 1243, 1320, 1564 (*gabonicum*); 0013, 0014, 0015, 1748, 1755, 1788, 1807, 1828, 1830, 1855, 1856, 1865 (*glabrum*); 16, 17, 18, 19, 20, 21, 23, 24, 26, 27, 29, 30, 187, 188, 189, 193, 194, 195, 220, 221, 228, 232, 291, 292, 299, 1113, 1118, 1131, 1153, 1184, 1191, 1197, 1206, 1210, 1233, 1273, 1299, 1336, 1451, 1725, 1726, 1731, 1741, 1758, 1770, 1772, 1795, 1807, 1827, 1840, 1854, 1865, 1892, 1894, 1900, 1913, 1928, 1944, 1970, 1999, 2083, 2100 (*suaveolens*)

Louis, A.M. 308, 550, 903, 310, 1415, 1450, 2987, 3119, 9082, 10916 (*suaveolens*)

Mann, G. 841 (*oliveri*)

Marshall, A.R. 1094, 1260 (*usambaricum*)

Martineau 318 (*oliveri*)

Maudoux, E. 584 (*suaveolens*)

MBG transect 1132 (*gabonicum*); 2242 (*suaveolens*)

M’Boungou, R. 506 (*suaveolens*)

McPherson, G.D. 13716, 13736, 13777, 15293, 15498 (*gabonicum*); 15501, 15802 (*glabrum*); 16812 (*littorale*); 21460 (*oliveri*); 13907, 13748, 13846 (*suaveolens*)

Meiren, J.J. van der 40 (*suaveolens*)

Merello, M.C. 1341 (*oliveri*)

Minkébé Series 294 (*suaveolens*)

Mitani, M. 10, 206 (*glabrum*)

Mortehan, M.G. 362 (*suaveolens*)

Mpandzou, A.L. 202, 1104 (*suaveolens*)

Mpom, B. 74, 249, 537, 573 (*suaveolens*)

Nemba, J. 291 (*suaveolens*)

Ngok Banak, L. 45 (*glabrum*)

Ngomo, D. 342 (*suaveolens*)

Nkongmeneck, B.-.A. 400 (*glabrum*)

Niangadouma, R. 22 (*gabonicum*)

Ogonovszky, M. 293 (*suaveolens*)

Oldeman, R.A.A. 856 (*oliveri*)

Oliveira, F. de 588 (*suaveolens*)

Peter, A. 17595, 18130 (*usambaricum*)

Philippe 83 (glabrum); 6 (*suaveolens*)

Reitsma, J.F. 1737, 2337, 2580, 2957 (*suaveolens*)

Reitsma, J.M. 1030, 1434, 1679 (*gabonicum*); 2173, 2206, 2337, 3478 (*suaveolens*)

Robin, R. 90 (*suaveolens*)

Ruffo, C.K. 2195 (*usambaricum*)

Schmidt, H.H. 1706 (*oliveri*)

Scouppe, M. 139 (*oliveri*)

Sense, S.R. 3238 (*usambaricum*)

Senterre, B. 697, 171 (*glabrum*); 784 (*suaveolens*)

Sita, P. 4045 (*gabonicum*); 3698 (*littorale*)

Sonké, B. 453, 5549, 5787 (*suaveolens*)

Sosef, M.S.M. 1684 (*suaveolens*)

Soyaux, H. 218 (*suaveolens*)

Steemers, B.J. 1 (*suaveolens*)

Stévart, T.O.B.E.B. 4655, 4764 (*suaveolens*)

Stone, J.R. 3169 (*gabonicum*)

Tailfer, Y. 24 (*suaveolens*)

Thijssen, M.T. 317 (*oliveri*)

Thomas, D.W. 5059 (*oliveri*); 725, 3349, 4878 (*suaveolens*)

Tisserant, R.P. 358, 677, 1172, 2204, 2573 (*suaveolens*)

Toelen 24 (*suaveolens*)

Toka, L. 179 (*suaveolens*)

Toussaint, L. 2113, 2118, 2203 (*suaveolens*)

van Andel, T.R. 3318, 3794, 4076 (*suaveolens*)

van Meer, P.P.C. 1795, 1228 (*suaveolens*)

Voorhoeve, A.G. 1073 (*oliveri*)

Wagemans, J. 966 (*suaveolens*)

Walters, G.M. 656 (*suaveolens*)

Webb, J. 390, 539 (*glabrum*)

White (series 1), L.J.T. 97 (*gabonicum*)

Wieringa, J.J. 5129 (*glabrum*); 2476, 8490 (*littorale*); 1395 (*suaveolens*)

Wilde de, J.J.F.E. 11433 (*gabonicum*); 8465 (*glabrum*); 11217 (*littorale*); 10083 (*oliveri*); 73, 101, 7940, 8674 (*suaveolens*)

Wilde de, W.J.J.O. 2133 2857 (*suaveolens*); 510 (*oliveri*)

Wilks, C.M. 2695 (*gabonicum*); 1544 (*suaveolens*)

Wit, P. 2111 (*suaveolens*)

Yalibanda, Y. 94/36 (*suaveolens*)

Yangakola, J-M. 111, 153 (*suaveolens*)

Zenker, G.A. 328, 1278, 1284, 1306, 1633, 2062, 2166, 2841, 3872, 3872a, 4435, 4739, 4841, 4896 (*suaveolens*)

## Supplementary Material

XML Treatment for Greenwayodendron

XML Treatment for Greenwayodendron gabonicum

XML Treatment for Greenwayodendron glabrum

XML Treatment for Greenwayodendron littorale

XML Treatment for Greenwayodendron oliveri

XML Treatment for Greenwayodendron suaveolens

XML Treatment for Greenwayodendron usambaricum
